# Diabetic Retinopathy Severity and Heart Failure Outcomes in Type 2 Diabetes Mellitus

**DOI:** 10.1111/1753-0407.70235

**Published:** 2026-07-02

**Authors:** Feng‐Ching Shen, Pei‐Kang Liu, Yi‐Chen Huang, Teng‐Hui Huang, Ming‐Yen Lin, Yi‐Hsueh Liu, Tien‐Chi Huang, Mei‐Chuan Kuo, Yi‐Wen Chiu, Shang‐Jyh Hwang, Yi‐Ting Lin, Ping‐Hsun Wu

**Affiliations:** ^1^ Graduate Institute of Clinical Medicine, College of Medicine, Kaohsiung Medical University Kaohsiung Taiwan; ^2^ Division of Nephrology, Department of Internal Medicine Kaohsiung Medical University Hospital, Kaohsiung Medical University Kaohsiung Taiwan; ^3^ Department of Ophthalmology Kaohsiung Medical University Hospital, Kaohsiung Medical University Kaohsiung Taiwan; ^4^ Faculty of Medicine College of Medicine, Kaohsiung Medical University Kaohsiung Taiwan; ^5^ Division of Cardiology, Department of Internal Medicine Kaohsiung Medical University Hospital, Kaohsiung Medical University Kaohsiung Taiwan; ^6^ Department of Internal Medicine Kaohsiung Municipal Siaogang Hospital Kaohsiung Taiwan; ^7^ Department of Family Medicine Kaohsiung Medical University Hospital Kaohsiung Taiwan; ^8^ Center for Big Data Research, Kaohsiung Medical University Kaohsiung Taiwan; ^9^ Research Center for Precision Environmental Medicine, Kaohsiung Medical University Kaohsiung Taiwan; ^10^ Biomedical Artificial Intelligence Academy, Kaohsiung Medical University Kaohsiung Taiwan; ^11^ Big Data Center, China Medical University Hospital, China Medical University Taichung Taiwan

**Keywords:** cardiac dysfunction, echocardiography cardiovascular outcomes, left ventricular systolic dysfunction, microvascular disease, risk stratification

## Abstract

**Background:**

The precise link between diabetic retinopathy and incident heart failure remains unclear, largely limited by a lack of echocardiographic data. This study evaluated how the clinical severity of retinopathy relates to incident heart failure and baseline cardiac function in individuals with type 2 diabetes.

**Methods:**

This retrospective cohort study (2005–2021) included adults with type 2 diabetes receiving structured management and color fundus imaging. Retinopathy was classified as absent, mild nonproliferative, or moderate‐to‐severe nonproliferative to proliferative. A specific subcohort received baseline transthoracic echocardiography. Relationships between cardiac function parameters and retinal damage were assessed using ordinal logistic regression with inverse probability weighting. Incident heart failure risk was determined utilizing Cox proportional hazards and Poisson regression models.

**Results:**

Among 21 773 participants, heart failure was independently associated with advanced retinopathy (OR, 1.23). After inverse probability weighting (IPW), associations with systolic dysfunction (unweighted OR, 1.96; IPW‐adjusted OR, 2.01) and diastolic dysfunction were significant, with the latter explicitly unmasked by IPW (unweighted OR, 1.08 vs. IPW‐adjusted OR, 1.17). The risk for incident heart failure escalated alongside progressive retinal damage. Compared to patients presenting without retinopathy, the adjusted incidence rate ratios were 1.64 for the mild group and 1.86 for those with moderate‐to‐severe or proliferative disease, an association partially mediated by renal function decline.

**Conclusions:**

Diabetic retinopathy severity associates with systolic and diastolic dysfunction and is an independent risk factor for incident heart failure in type 2 diabetes. Routine retinal evaluation serves as a practical clinical indicator for assessing systemic microvascular damage and cardiovascular risk.

## Highlights

1. Worsening diabetic retinal disease serves as an independent risk factor for incident heart failure in the type 2 diabetic population.
2. A baseline history of heart failure and impaired left ventricular function (both systolic and diastolic) was independently associated with more severe retinopathy.
3. Including retinal severity metrics in cardiovascular risk profiling informs early surveillance and therapeutic strategies for heart failure.

## Graphical abstract

In type 2 diabetes, worsening diabetic retinopathy is associated with both systolic and diastolic dysfunction and independently predicts incident heart failure. Routine retinal evaluation may help identify patients at increased microvascular and cardiovascular risk.

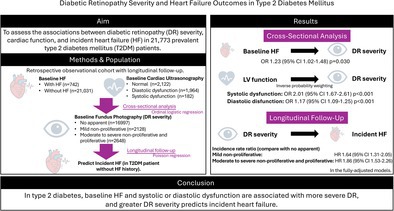

## 1 Introduction

Retinal microvascular damage, commonly presenting as diabetic retinopathy (DR), frequently co‐occurs with systemic cardiovascular conditions [1]. Individuals progressing to proliferative stages of the disease demonstrate higher incidences of coronary artery pathology and cardiovascular‐related mortality [2]. Furthermore, the presence of retinal manifestations in the diabetic population correlates with a greater probability of subsequent heart failure [3]. Clinical observations also link this retinal impairment directly to diminished resting myocardial performance, encompassing abnormalities in both the filling and pumping phases of the cardiac cycle [4].

The clinical correlation between retinal disease and cardiac dysfunction is widely recognized. Because retinal changes reflect broader systemic microvascular damage, these ocular findings often parallel negative structural remodeling in the heart, suggesting an underlying susceptibility to diabetic cardiomyopathy and acute heart failure events [5, 6]. Despite this established background, the extent to which advancing stages of retinal damage multiply the probability of acute heart failure remains a subject of ongoing debate. Moreover, the exact correlation linking retinal disease progression to specific left ventricular functional parameters lacks detailed investigation, presenting a need to better define how ocular grading aligns with echocardiographic findings in this patient population.

To address these gaps, the current research investigates how the clinical grade of retinal disease influences the future probability of developing heart failure over a given observational timeframe. Baseline analyses examined the relationship between heart failure history, inverse probability weighting‐adjusted left ventricular function, and retinopathy severity. The results of this analysis aim to clarify the clinical dynamics connecting microvascular eye complications to diabetic cardiac dysfunction, thereby supporting more precise risk assessment and clinical care for vulnerable individuals.

## 2 Methods

### 2.1 Study Design and Data Source

This retrospective cohort analysis extracted clinical records from the Kaohsiung Medical University Hospital Research Database (KMUHRD). We evaluated the cross‐sectional relationship relating baseline cardiac function and prior heart failure to the stage of retinal disease. A subsequent longitudinal assessment evaluated how baseline retinal status predicts future acute heart failure. The source database aggregates health information from patients receiving care at Kaohsiung Medical University Hospital and associated regional centers from 2005 through 2021. Ethics approval was granted by the institutional review board at our center (KMUHIRB‐E(II)‐20 220 286). All individuals or their legal proxies provided written informed consent prior to inclusion, and the research protocol adhered to established ethical standards.

### 2.2 Study Population

Eligibility for this study required participants to be adults diagnosed with type 2 diabetes who were active in the Taiwan Diabetes Mellitus Pay‐for‐Performance (DM P4P) care program. To qualify for inclusion, subjects needed at least two clinic visits coded for diabetes within the preceding 90 days, and the index visit required diabetes as the main clinical diagnosis. Disease classification relied on standard diagnostic codes (ICD‐9‐CM 250.x0 or 250.x2; ICD‐10‐CM E11.x). Any subject with a documented history of type 1 diabetes (ICD‐9‐CM 250.x1/250.x3 or ICD‐10‐CM E10.x) was removed from the cohort.

### 2.3 Clinical Variables and Covariates

We obtained baseline subject characteristics including physical measurements (body mass index, systolic blood pressure, age, sex), coexisting conditions (chronic obstructive pulmonary disease, atrial fibrillation, coronary artery disease, hypertension), and laboratory findings (glycated hemoglobin, low‐density lipoprotein cholesterol, and estimated glomerular filtration rate calculated by the CKD–EPI formula). Pharmacological data covered prescriptions for statins, beta‐blockers, renin–angiotensin system antagonists, sodium‐glucose cotransporter‐2 (SGLT2) inhibitors, glucagon‐like peptide‐1 (GLP‐1) receptor agonists, and ocular anti‐vascular endothelial growth factor injections. Albuminuria was evaluated using the urine albumin‐to‐creatinine ratio (UACR) and classified into three clinical stages: A1 (< 30 mg/g, normal to mildly increased), A2 (30–299 mg/g, moderately increased), and A3 (≥ 300 mg/g, severely increased). A sensitivity analysis was further performed to evaluate the impact of insulin use. Retinal status was separated into five clinical stages: no visible disease, mild nonproliferative changes, moderate nonproliferative changes, severe nonproliferative changes, and proliferative disease. The day patients underwent color fundus imaging served as the index date.

Cardiac function was assessed by specialists relying on standardized echocardiographic measures. Left ventricular systolic parameters were determined according to ASE protocols; specialists applied the modified Simpson's biplane approach from two‐ and four‐chamber apical views to derive ventricular volumes and the ejection fraction [7]. The assessment of diastolic function followed the 2016 joint recommendations from the ASE and EACVI. This diagnostic algorithm integrated several specific parameters: the ratio of early to late transmitral flow, early diastolic velocities at the septal and lateral mitral annulus, the average ratio of transmitral to annular early diastolic velocities, indexed left atrial volume, and the peak velocity of the tricuspid regurgitation jet. Together, these metrics categorized diastolic impairment and estimated intracardiac filling pressures [8]. The main endpoint of the study was an occurrence of acute heart failure. This was defined as any inpatient admission or emergency department presentation where heart failure served as the primary clinical diagnosis, confirmed by corresponding diagnostic codes (ICD‐9‐CM 428.x or ICD‐10‐CM I50.x).

### 2.4 Assessment of Diabetic Retinopathy and Cardiac Function

We identified individuals with type 2 diabetes who had both initial echocardiographic and fundoscopic evaluations documented in the KMUHRD as part of the Taiwan DM P4P care program [9]. Recruitment occurred via clinics associated with this network, and multivariable regression techniques controlled for variations in demographic and clinical profiles. Prior heart failure was defined by its presence as a main diagnosis during at least two outpatient consultations or any single inpatient or emergency admission. Cardiologists interpreted all ultrasound data. Retinal photography was performed using a nonmydriatic imaging device (Canon CRDGi equipped with a 20‐diopter SLR back, Canon, Japan), which was evaluated subsequently by trained endocrinologists. To verify grading consistency, an independent ophthalmologist reviewed a 240‐patient validation sample. Inter‐rater reliability, calculated via a quadratic weighted Cohen's kappa, reached 0.767, reflecting a 72% overall agreement. Retinopathy diagnoses relied on the identification of specific pathological features based on the 2019 practice patterns issued by the American Academy of Ophthalmology [10]. Heart failure was documented using established diagnostic codes, specifically ICD‐10‐CM (I09.9, I11.0, I13.0, I13.2, I25.5, I42.0, I42.5–I42.9, I43.x, I50.x, P29.0) and ICD‐9‐CM (398.91, 402.01, 402.11, 402.91, 404.01, 404.03, 404.11, 404.13, 404.91, 404.93, 428). Renal function was estimated at the index date using serum creatinine inputted into the 2021 CKD–EPI formula [11]. Kidney function stages were defined by standard thresholds: ≥ 90 (G1), 60–89 (G2), 30–59 (G3), 15–29 (G4), and < 15 (G5) mL/min/1.73 m^2^.

### 2.5 Study Size and Bias

The final analysis cohort consisted of participants active in the Taiwan DM P4P care program [9, 12] whose medical records in the database contained parallel echocardiographic and retinal imaging results. We managed heterogeneity in the clinical and demographic characteristics of this opportunistically sampled clinic population by applying multivariable regression adjustments. While individual clinicians performed the cardiac and retinal examinations, we maintained diagnostic uniformity and reduced potential reporting bias by requiring the use of structured evaluation protocols.

### 2.6 Quantitative Variables

To analyze how retinal disease progression relates to future acute heart failure, patients were divided into three progressive groups: absence of visible retinopathy, mild nonproliferative changes, and an advanced group combining moderate/severe nonproliferative with proliferative disease. This 3‐grade classification was based on clinical and statistical considerations. Clinically, the moderate‐to‐severe and proliferative stages represent a threshold of advanced microvascular impairment requiring intensive management. Statistically, collapsing the most advanced grades ensured sufficient event numbers to maintain adequate power and stabilize the regression estimates. The final ocular grade assigned to each patient corresponded to the eye with the worse clinical presentation when bilateral imaging was available. Initial cardiac function was classified by specialists as normal, displaying diastolic impairment, or displaying systolic impairment. These classifications followed strict adherence to joint ASE/EACVI recommendations, evaluating the ejection fraction with the biplane Simpson method for systole and applying the established 2016 diagnostic algorithm for diastole.

### 2.7 Statistical Analysis

Baseline participant variables were evaluated across different categories of retinal disease severity and cardiac left ventricular performance. Continuous data meeting normality criteria were compared using one‐way ANOVA (analysis of variance), while categorical differences were analyzed with chi‐squared statistics. To determine how the stages of retinal damage correlate with existing heart failure, cardiac function parameters, and the administration of diuretics (used as a clinical surrogate for heart failure severity), we applied ordinal logistic regression. The proportional odds assumption required for this regression approach was confirmed via the test of parallel lines, which returned nonsignificant results, confirming the suitability of this method for the graded retinal classifications.

In the subgroup of patients without prior cardiac failure events, the hazard of developing future acute heart failure relative to retinal disease stage was calculated using Cox proportional hazards models. The proportional hazards assumption was confirmed. We mapped the time‐to‐event probability for new heart failure diagnoses among the ocular severity cohorts using the Kaplan–Meier estimator, and differences between these groups were tested with the log‐rank method. Incidence rate ratios comparing incident heart failure across the varying degrees of retinal damage were derived via Poisson regression analysis. Furthermore, we conducted subgroup and interaction analyses using likelihood ratio tests. Mediation analysis was performed to quantify the extent to which renal factors, specifically estimated glomerular filtration rate (eGFR) and urine albumin‐to‐creatinine ratio (UACR) stage, mediated the association between DR and heart failure risk. Furthermore, to mitigate potential selection bias within the opportunistically sampled echocardiography subcohort (*n* = 4268), an inverse probability weighting (IPW) approach was employed. Propensity scores for undergoing echocardiography were estimated using a multivariable logistic regression model that incorporated a comprehensive set of baseline covariates: demographics (age and sex), physical measurements (systolic blood pressure and body mass index), comorbidities (hypertension, coronary artery disease, atrial fibrillation, and chronic obstructive pulmonary disease), current medications (beta‐blockers, ACEI/ARB, statins, SGLT2 inhibitors, and GLP‐1 receptor agonists), and laboratory data (low‐density lipoprotein cholesterol, glycated hemoglobin, and estimated glomerular filtration rate calculated by the CKD–EPI formula). Stabilized inverse probability weights were derived from the predicted probability of undergoing echocardiography in the full cohort (*N* = 21 773), calculated via a multivariable logistic regression model incorporating baseline demographic and clinical covariates. These weights were subsequently applied to the ordinal logistic regression models assessing the relationship between cardiac function and retinopathy severity. Data analysis was carried out using Jamovi version 2.4.14 (https://www.jamovi.org/) and SAS software, version 9.4 (SAS Institute, Cary, North Carolina, USA).

## 3 Results

### 3.1 Study Flow Diagram and Baseline Characteristics

This analysis included 21 773 individuals with type 2 diabetes from the Taiwan DM P4P care program. All participants possessed retinal imaging data, and a subcohort of 4268 underwent baseline echocardiography. We evaluated how clinical heart failure status and cardiac sonographic parameters align with retinal disease stages and forecast new‐onset heart failure (Figure S1).

Table 1 outlines initial participant profiles based on retinal grading. Subjects exhibiting advanced retinal damage tended to be older, female, and hypertensive, with lower body mass indices. They presented higher rates of coronary artery disease, preexisting heart failure, and advanced renal decline, alongside increased prescriptions for beta‐blockers, renin‐angiotensin‐aldosterone system inhibitors, and anti‐VEGF intravitreal therapies. Worse retinal grades also aligned with poorer glycemic control and reduced estimated glomerular filtration rates.

**TABLE 1:** Baseline characteristics by diabetic retinopathy severity.

	No apparent (*n* = 16 997)	Mild nonproliferative (*n* = 2128)	Moderate to severe nonproliferative and proliferative (*n* = 2648)	*p*	SMD (mild vs no apparent)	SMD (moderate to severe nonproliferative and proliferative vs no apparent)
Baseline demographic						
Age (years)	59.0 ± 14.0	60.9 ± 12.6	59.2 ± 12.3	< 0.001	0.141	0.017
Male gender, *n* (%)	8991 (52.9)	1073 (50.4)	1355 (51.2)	0.037	0.050	0.035
Systolic blood pressure (mmHg)	132.8 ± 14.0	135.0 ± 14.6	138.2 ± 16.1	< 0.001	0.149	0.354
Body mass index (kg/m^2^)	26.3 ± 4.5	25.9 ± 4.2	25.8 ± 4.3	< 0.001	0.105	0.110
Comorbidities (*n*, %)						
eGFR category (mL/min/1.73m^2^)				< 0.001		
G1 (≥ 90)	10 380 (61.3)	1226 (58.0)	1304 (49.3)		0.067	0.243
G2 (60–89)	4374 (25.8)	501 (23.7)	701 (26.5)		0.049	0.016
G3 (30–59)	1750 (10.3)	294 (13.9)	439 (16.6)		0.111	0.185
G4 + 5 (< 30)	441 (2.6)	92 (4.4)	199 (7.6)		0.098	0.229
Hypertension	10 455 (61.5)	1509 (70.9)	2003 (75.6)	< 0.001	0.200	0.308
Coronary artery disease	1423 (8.4)	153 (7.2)	267 (10.1)	0.001	0.044	0.059
Heart failure	544 (3.2)	59 (2.8)	139 (5.2)	< 0.001	0.023	0.100
Atrial fibrillation	290 (1.7)	20 (0.9)	42 (1.6)	0.030	0.067	0.009
Hyperlipidemia	6353 (37.4)	781 (36.7)	971 (36.7)	0.681	0.014	0.015
Chronic obstructive pulmonary disease	419 (2.5)	45 (2.1%)	47 (1.8)	0.070	0.023	0.048
Medications (*n*, %)						
Beta blocker	3521 (20.7)	408 (19.2)	626 (23.6)	< 0.001	0.039	0.070
ACEI/ARB	5975 (35.2)	854 (40.1)	1263 (47.7)	< 0.001	0.103	0.257
Statin	6482 (38.1)	787 (37.0)	1046 (39.5)	0.196	0.024	0.028
IVI Anti‐VEGF	15 (0.1)	4 (0.2)	25 (0.2)	< 0.001	0.026	0.026
SGLT2 inhibitor	822 (4.8)	82 (3.9)	84 (3.2)	< 0.001	0.048	0.085
Glucagon‐like peptide‐1 agonist	124 (0.7)	26 (1.2)	26 (1.0)	0.033	0.050	0.027
Laboratory data						
Low‐density lipoprotein (mg/dl)	105.9 ± 37.7	107.1 ± 40.1	106.6 ± 39.8	0.298	0.032	0.018
Glycated hemoglobin (%)	8.2 ± 2.3	8.6 ± 2.2	9.0 ± 2.4	< 0.001	0.168	0.339
eGFR (mL/min/1.73 m^2^)	90.3 ± 24.9	86.4 ± 26.8	81.7 ± 30.1	< 0.001	0.150	0.312
UACR category (*n* = 16 529)				< 0.001		
Normal to mildly increased	6641 (50.2)	643 (45.7)	459 (24.2)		0.091	0.560
Moderately increased	2452 (18.5)	349 (24.8)	412 (21.7)		0.152	0.079
Severely increased	4129 (31.2)	416 (29.5)	1028 (54.1)		0.037	0.476

*Note:* The data are expressed as mean ± standard deviation unless otherwise stated. eGFR is calculated by the CKD–EPI (Chronic Kidney Disease Epidemiology Collaboration) equation. An absolute standardized mean difference greater than 0.10 was considered indicative of meaningful imbalance.

Abbreviations: ACEI, angiotensin‐converting enzyme inhibitor; ARB, angiotensin II receptor blocker; eGFR, estimated glomerular filtration rate; IVI, intravitreal injection; NPDR, nonproliferative diabetic retinopathy; PDR, proliferative diabetic retinopathy; SGLT2, sodium‐glucose cotransporter 2; SMD, standardized mean difference; UACR, urine albumin‐to‐creatinine ratio; VEGF, vascular endothelial growth factor.

Regarding echocardiographic profiles (Table S1), individuals displaying systolic impairment were predominantly older males with higher frequencies of arrhythmias, hypertension, and ischemic heart disease. This subgroup also demonstrated worse renal parameters, higher glycated hemoglobin, greater statin administration, and more severe degrees of retinal microvascular damage. Furthermore, a baseline comparison revealed that the overall echocardiographic subcohort was older and had more cardiovascular comorbidities than the nonechocardiographic population, reflecting expected clinical indication bias (Table S2). These baseline differences were rigorously accounted for in our subsequent multivariable adjustment models.

### 3.2 Association Between Heart Failure Status and Diabetic Retinopathy Severity

Table 2 details the ordinal logistic regression evaluating prior heart failure against retinal disease stages. Following full covariate adjustment, a clinical history of heart failure correlated positively with worse retinal grading (odds ratio [OR], 1.23; 95% confidence interval [CI], 1.02–1.48; *p* = 0.030). Diuretic administration, reflecting fluid overload severity, also indicated a strong positive relationship with advanced ocular disease (OR, 1.84; 95% CI, 1.24–2.75; *p* = 0.003) (Table S3). This primary correlation remained stable even among individuals never treated with anti‐VEGF injections (OR, 1.22; *p* = 0.040) (Table S4).

**TABLE 2:** Association between heart failure and diabetic retinopathy severity (*n* = 21 773).

	Odds ratio (95% confidence interval)	*p*
Diabetic retinopathy severity		
Model 1	1.51 (1.27–1.79)	< 0.001
Model 2	1.48 (1.23–1.77)	< 0.001
Model 3	1.47 (1.22–1.76)	< 0.001
Model 4	1.23 (1.02–1.48)	0.030

*Note:* Model 1: Age, gender, systolic blood pressure, body mass index. Model 2: Model 1 + comorbidity (hypertension, coronary artery disease, atrial fibrillation, chronic obstructive pulmonary disease). Model 3: Model 2 + medications (angiotensin converting enzyme inhibitor/angiotensin II receptor blocker, beta blocker, statin, SGLT2 inhibitors, GLP‐1 receptor agonists). Model 4: Model 3 + laboratory data (low‐density lipoprotein‐cholesterol, glycated hemoglobin, estimated glomerular filtration rate measured by CKD–EPI (Chronic Kidney Disease Epidemiology Collaboration)).

Abbreviations: GLP‐1, glucagon‐like peptide‐1; SGLT2, sodium‐glucose cotransporter 2.

Further stratification revealed that the link between clinical heart failure and severe retinal damage was highly pronounced in the severe kidney disease subgroup (eGFR stages G4‐G5; OR, 2.13; *p* = 0.010), but lost statistical significance in those with preserved or mildly reduced renal function (eGFR G1‐G3; OR, 1.26; *p* = 0.260) (Table S5 and Figure S2). Additional subgroup evaluations (Figure S3) confirmed significant associations for patients under 65 years old (OR, 1.37; *p* = 0.028) and those with coexisting hypertension (OR, 1.23; *p* = 0.010).

### 3.3 Association Between Left Ventricular Function and Diabetic Retinopathy Severity

To rigorously account for clinical indication bias within the opportunistically sampled echocardiography subcohort, we employed an IPW analysis as our primary evaluation. Table 3 presents both the unweighted and IPW‐adjusted regression outputs side by side, balancing the baseline characteristics to reflect the broader study population while allowing for direct comparison.

**TABLE 3:** Unweighted and inverse probability weighting‐adjusted associations of left ventricular dysfunction with diabetic retinopathy severity.

Overall cohort	Diabetic retinopathy severity							
	Systolic dysfunction (*N* = 182)				Diastolic dysfunction (*N* = 1964)			
	Unweighted		IPW‐adjusted		Unweighted		IPW‐adjusted	
	Odds ratio (95% CI)	*p*	Odds ratio (95% CI)	*p*	Odds ratio (95% CI)	*p*	Odds ratio (95% CI)	*p*
Model 1	2.32 (1.65–3.21)	< 0.001	2.31 (1.93–2.76)	< 0.001	1.17 (1.001–1.36)	0.049	1.25 (1.17–1.34)	< 0.001
Model 2	2.31 (1.65–3.22)	< 0.001	2.33 (1.94–2.79)	< 0.001	1.15 (0.98–1.34)	0.081	1.23 (1.15–1.32)	< 0.001
Model 3	2.30 (1.63–3.20)	< 0.001	2.29 (1.90–2.74)	< 0.001	1.13 (0.97–1.32)	0.121	1.21 (1.13–1.30)	< 0.001
Model 4	1.96 (1.39–2.75)	< 0.001	2.01 (1.67–2.41)	< 0.001	1.08 (0.92–1.27)	0.323	1.17 (1.09–1.25)	< 0.001

Without CAD	Systolic dysfunction (*N* = 60)				Diastolic dysfunction (*N* = 1425)			
	Unweighted		IPW‐adjusted		Unweighted		IPW‐adjusted	
	Odds ratio (95% CI)	*p*	Odds ratio (95% CI)	*p*	Odds ratio (95% CI)	*p*	Odds ratio (95% CI)	*p*
Model 1	2.47 (1.60–3.77)	< 0.001	2.13 (1.73–2.61)	< 0.001	1.16 (0.99–1.37)	0.074	1.28 (1.19–1.37)	< 0.001
Model 2	2.47 (1.60–3.78)	< 0.001	2.14 (1.74–2.63)	< 0.001	1.14 (0.96–1.34)	0.128	1.25 (1.16–1.34)	< 0.001
Model 3	1.96 (1.20–3.11)	0.005	2.08 (1.69–2.55)	< 0.001	1.10 (0.92–1.32)	0.291	1.24 (1.15–1.33)	< 0.001
Model 4	1.67 (1.03–2.67)	0.035	1.85 (1.50–2.27)	< 0.001	1.06 (0.88–1.27)	0.542	1.19 (1.10–1.28)	< 0.001

*Note:* Reference group: normal left ventricular function. Unweighted odds ratios were calculated using standard ordinal logistic regression. IPW‐adjusted odds ratios were estimated using inverse probability weighted ordinal logistic regression to account for nonrandom selection into the echocardiography subgroup. Stabilized inverse probability weights were derived from the predicted probability of undergoing echocardiography in the full cohort. Model 1: Age, gender, systolic blood pressure, body mass index. Model 2: Model 1 + comorbidity (hypertension, coronary artery disease, atrial fibrillation, chronic obstructive pulmonary disease). Model 3: Model 2 + medications (angiotensin converting enzyme inhibitor/angiotensin II receptor blocker, beta blocker, statin, SGLT2 inhibitors, GLP‐1 receptor agonists). Model 4: Model 3 + laboratory data (low‐density lipoprotein‐cholesterol, glycated hemoglobin, estimated glomerular filtration rate measured by CKD–EPI (Chronic Kidney Disease Epidemiology Collaboration)).

Abbreviations: CAD, coronary artery disease; CI, confidence interval; CKD–EPI, Chronic Kidney Disease Epidemiology Collaboration; GLP‐1, glucagon‐like peptide‐1; IPW, inverse probability weighting; SGLT2, sodium‐glucose cotransporter 2.

In the fully adjusted IPW model, systolic impairment correlated with a doubled risk of worse retinal disease compared to normal cardiac function (IPW‐adjusted OR, 2.01; 95% CI, 1.67–2.41; *p* < 0.001). Furthermore, correcting for selection bias revealed a significant, independent association between diastolic dysfunction and advanced retinopathy grading (IPW‐adjusted OR, 1.17; 95% CI, 1.09–1.25; *p* < 0.001). Importantly, this dual association with both systolic and diastolic impairments persisted even after excluding patients with documented coronary artery disease (Systolic OR, 1.85; Diastolic OR, 1.19; both *p* < 0.001).

For comparison, the unweighted regression analysis—which was inherently subject to selection bias and initially obscured the significance of diastolic dysfunction—is provided alongside the adjusted data in Table 3. Figure S4 illustrates the visual distribution of retinal severity across varying categories of preserved, mildly reduced, and reduced ejection fractions.

### 3.4 Prediction of Incident Heart Failure by Severity of Diabetic Retinopathy

For participants without pre‐existing heart failure, time‐to‐event analysis (Figure S5) demonstrated a clear, stepwise escalation in new‐onset heart failure as baseline retinal disease worsened (log‐rank *p* < 0.001). Subjects presenting with moderate‐to‐severe nonproliferative or proliferative changes experienced the highest event frequencies.

This graded risk pattern remained consistent across most evaluated demographic and clinical subgroups (Figure S6). However, the predictive value of retinal damage on incident heart failure weakened statistically among normotensive individuals (*p* = 0.054) and those with severe renal impairment (eGFR G4‐5; *p* = 0.160).

Table 4 quantifies these future risks. Event frequencies rose from 6.48 per 1000 person‐years for individuals with normal retinas to 18.54 per 1000 person‐years for those with the most advanced ocular disease. Adjusting for all covariates, the incidence rate ratios relative to the normal‐retina baseline were 1.64 (95% CI, 1.31–2.05) for the mild nonproliferative group and 1.86 (95% CI, 1.53–2.26) for the advanced group. Fully adjusted proportional hazards models verified this escalating vulnerability, yielding hazard ratios of 1.64 (95% CI, 1.31–2.05) and 1.85 (95% CI, 1.53–2.25) for the mild and advanced retinal disease categories, respectively.

**TABLE 4:** Incident heart failure according to diabetic retinopathy severity (*n* = 21 031).

	No apparent (*n* = 16 453)	Mild nonproliferative (*n* = 2069)	Moderate to severe nonproliferative and proliferative (*n* = 2509)	*p* for trend
Incident heart failure (*n*)	359	100	168	
Person‐year	55380.7	7583.42	9059.64	
Incidence rate (per 1000 person‐year)	6.48 (5.83–7.19)	13.19 (10.53–16.53)	18.54 (15.27–22.52)	
Crude incidence rate ratio	Reference	2.03 (1.63–2.54)	2.86 (2.38–3.44)	< 0.001
Adjusted incidence rate ratio	Reference	1.64 (1.31–2.05)	1.86 (1.53–2.26)	< 0.001
Hazard ratio				
Model 1	Reference	1.79 (1.49–2.15)	2.88 (2.47–3.35)	< 0.001
Model 2	Reference	1.79 (1.49–2.15)	2.74 (2.35–3.18)	< 0.001
Model 3	Reference	1.88 (1.50–2.35)	2.72 (2.25–3.28)	< 0.001
Model 4	Reference	1.64 (1.31–2.05)	1.85 (1.53–2.25)	< 0.001

*Note:* Participants without pre‐existing heart failure were included in this analysis.

The data are expressed as ratio (95% confidence interval) unless otherwise stated.

Model 1: Age, gender, systolic blood pressure, body mass index.

Model 2: Model 1 + comorbidity (hypertension, coronary artery disease, atrial fibrillation, chronic obstructive pulmonary disease).

Model 3: Model 2 + medications (angiotensin converting enzyme inhibitor/angiotensin II receptor blocker, beta blocker, statin, SGLT2 inhibitors, GLP‐1 receptor agonists).

Model 4: Model 3 + laboratory data (low‐density lipoprotein‐cholesterol, glycated hemoglobin, estimated glomerular filtration rate measured by CKD–EPI (Chronic Kidney Disease Epidemiology Collaboration)).

Abbreviations: GLP‐1, glucagon‐like peptide‐1; SGLT2, sodium‐glucose cotransporter 2.

Furthermore, an analysis utilizing the original 5‐grade retinal classification confirmed a highly significant, linear dose–response relationship between worsening diabetic retinopathy and incident heart failure risk (*p* for trend < 0.001; Table S6).

Furthermore, we performed a stratified sensitivity analysis by insulin use status, given that insulin use may reflect underlying diabetes severity and longer disease duration. As shown in Table S7, among patients not receiving insulin, the graded increase in incident heart failure risk across diabetic retinopathy severity remained robust (*p* for trend < 0.001).

Finally, interaction and mediation analyses were performed to further contextualize these longitudinal findings. As shown in Figure S7, the graded association between advanced DR and incident heart failure remained highly consistent, with no significant interaction observed by hypertension (*p* for interaction > 0.05). Exploratory mediation analysis (Table S8) suggested that eGFR served as a significant partial mediator in the association between DR and AHF (*p* < 0.001 for indirect effects across all stages). While UACR stage also demonstrated a significant mediating effect in the advanced retinopathy group (*p* < 0.001), it did not significantly mediate the pathway for mild nonproliferative DR (*p* = 0.234).

## 4 Discussion

Our analysis establishes that advancing stages of retinal disease serve as a standalone predictor for future acute heart failure. When matched against subjects lacking visible retinal damage, the incidence rates for acute heart failure were 1.64 times higher in the mild NPDR group and 1.86 times higher in the moderate‐to‐severe NPDR and PDR cohort. Conversely, pre‐existing cardiac impairment was independently associated with microvascular ocular damage; specifically, a documented clinical history of heart failure (OR, 1.23) and impaired left ventricular function (including both systolic and diastolic dysfunctions) correlated independently with worse retinal disease grades.

Retinal evaluation provides a clinical window into the extent of systemic vascular pathology, as both ocular and cardiac complications in diabetes share underlying physiological pathways [13]. The progression of diabetes‐related cardiac impairment involves both large‐vessel and small‐vessel changes [14], often manifesting as myocardial ischemia [15] and primary diabetic cardiomyopathy [16]. Myocardial ischemia is driven by endothelial impairment and local inflammation, which accelerate microvascular atherosclerotic changes [17, 18]. Concurrently, diabetic cardiomyopathy is characterized by structural remodeling, fibrotic deposition, and microvascular injury, mechanisms closely tied to oxidative stress and impaired insulin response [14, 17]. Over time, these structural alterations lead to reduced systolic function and overt clinical heart failure [19]. Retinal microangiopathy may reflect a broader systemic microvascular phenotype that extends to the myocardium [3]. Chronic hyperglycemia promotes the accumulation of advanced glycation end‐products and reactive oxygen species, reduces nitric oxide bioavailability, and contributes to diffuse endothelial dysfunction [20]. In the heart, this process may manifest as coronary microvascular dysfunction, which has been linked to microvascular rarefaction, myocardial fibrosis, chronic subendocardial ischemia, increased myocardial stiffness, and impaired contractility [21]. Accordingly, severe diabetic retinopathy may serve as a clinically accessible marker of concurrent cardiac microcirculatory injury and may help explain the higher risk of heart failure observed in patients with advanced retinal disease.

Existing literature presents conflicting data regarding the connection linking retinal damage to large‐vessel conditions like coronary artery pathology. Certain investigations report that patients exhibiting retinal disease face greater rates of all‐cause mortality, broader cardiovascular conditions, and ischemic heart events [22]. In contrast, other trials found no definitive correlation connecting retinal damage to the onset of myocardial infarction or coronary blockages [23, 24]. From a histological perspective, evidence indicates that the specific arteriolar modifications seen in the diabetic retina differ from typical atherosclerotic plaques [25].

While the relationship connecting ocular disease to macrovascular events lacks consensus, the correlation between retinal grading and future heart failure is more robust. Retinal degradation functions as a proxy for widespread microvascular damage and predicts heart failure independently, even among populations lacking prior coronary artery pathology [26]. This supports the hypothesis that shared microcirculatory injury and microangiopathy simultaneously compromise both cardiac and ocular tissues [27]. The shared pathophysiological axis was further elucidated by our exploratory mediation analysis, which confirmed that renal function decline (specifically eGFR and UACR stage) acts as a significant partial mediator in the DR–HF pathway. However, the retention of a strong direct effect indicates that advanced diabetic retinopathy is not merely a bystander of renal or glycemic decline, but an independent marker of systemic microvascular vulnerability driving heart failure.

The administration of diuretics, a common clinical marker for fluid overload and heart failure severity, correlated positively with worse retinal disease grades in this analysis. In standard practice, escalating diuretic requirements reflect more difficult‐to‐manage congestion and edema among individuals with failing hearts [28]. Previous literature confirms that relying on these medications corresponds to a higher likelihood of hospital admissions and cardiovascular‐related death [29].

The link between compromised cardiac status and severe retinal microvascular changes was most apparent among participants exhibiting lower eGFR, suggesting that diabetic ocular and renal complications share underlying pathological pathways. These observations align with established epidemiological data where extensive retinal abnormalities occur frequently alongside severe chronic kidney disease [30]. Furthermore, worsening ocular microvascular disease historically tracks with deteriorating glomerular filtration, albuminuria, and the eventual transition to end‐stage renal failure [31, 32, 33].

Clinical retinal grading may reflect the severity and heterogeneity of left ventricular dysfunction in type 2 diabetes [3, 5]. Prior studies have linked diabetic retinopathy to adverse cardiac structure and function as well as a higher risk of incident heart failure, supporting the concept that retinal disease captures a broad spectrum of myocardial involvement [6]. In our initial unweighted analysis, the independent association appeared stronger for systolic dysfunction than for diastolic dysfunction. However, because the echocardiography subgroup was selected opportunistically rather than randomly, that analysis was susceptible to clinical indication bias, with overrepresentation of older individuals and those with a heavier burden of cardiovascular comorbidities. After applying inverse probability weighting to better approximate the source population of patients with type 2 diabetes, advanced retinopathy remained independently associated with both systolic and diastolic dysfunction. This shift suggests that the unweighted analysis may have underestimated the broader relationship between retinal microvascular disease and cardiac dysfunction, particularly for diastolic abnormalities, which may be less likely to be recognized in a clinically selected subgroup.

A biologically plausible explanation is that diabetic cardiomyopathy is heterogeneous and may manifest as distinct phenotypes dominated by either diastolic or systolic dysfunction [34]. This interpretation is further supported by recent evidence from the PROSE–HFpEF study, which highlighted systemic microvascular dysfunction across organs in heart failure with preserved ejection fraction and suggests that myocardial dysfunction may represent part of a broader multisystem microvascular process [35]. Chronic hyperglycemia promotes the accumulation of advanced glycation end‐products and reactive oxygen species, reduces nitric oxide bioavailability, and contributes to diffuse endothelial dysfunction [20]. In the myocardium, this process may manifest as coronary microvascular dysfunction, which has been linked to microvascular rarefaction, myocardial fibrosis, chronic subendocardial ischemia, increased myocardial stiffness, and impaired contractility [19, 21]. Because the retinal and coronary microcirculations share key features of small‐vessel beds, both may be particularly susceptible to hyperglycemia‐related microvascular injury [20]. Taken together, these findings suggest that severe diabetic retinopathy is not only associated with advanced contractile impairment but may also serve as a clinically accessible marker of the broader continuum of diabetic cardiac dysfunction, spanning both diastolic and systolic phenotypes.

Several constraints affect the interpretation of these findings. The retrospective nature of this cohort analysis restricts the ability to infer direct causality regarding retinal damage and new‐onset heart failure, meaning the results indicate correlations rather than cause‐and‐effect mechanisms. Furthermore, deriving clinical endpoints from electronic medical databases and billing codes carries an inherent risk of misclassification if charting inaccuracies or omissions occurred. The dataset also did not capture fine details regarding the length of time patients had diabetes or their compliance with prescribed medications, leaving room for unmeasured confounding variables. Specifically, because administrative EMR databases rely on the first recorded clinical encounter, ascertaining the true biological duration of type 2 diabetes was unfeasible. To partially mitigate this limitation, our primary models adjusted for clinical variables related to cumulative diabetes burden, including eGFR, glycated hemoglobin, and macrovascular comorbidities. In addition, because insulin therapy commonly reflects longer‐standing and more advanced diabetes in observational cohorts, we performed a stratified sensitivity analysis by insulin use. The association between diabetic retinopathy severity and incident heart failure remained robust across strata, supporting the stability of the main findings despite incomplete capture of true diabetes duration. Another constraint is that the echocardiographic subcohort was smaller in scale (*n* = 4268) and formed through opportunistic sampling, which introduces potential selection bias. As demonstrated in Table S2, this subcohort is inherently subject to clinical indication bias, as these patients presented with higher baseline cardiovascular risks and more comorbidities than the general study population. Although our multivariable models rigorously adjusted for these baseline covariates to ensure statistical robustness, extrapolating these subcohort findings to the broader, asymptomatic diabetic population should be approached with caution. Moreover, while independent validation showed adequate agreement between reviewers for the ocular photographs, the main ultrasound and retinal evaluations were carried out by one clinician, restricting the broader applicability of the results. In addition, our study was not designed to directly evaluate the myocardial mechanisms underlying these associations. We lacked direct assessments of myocardial perfusion, fibrosis, strain, and circulating biomarkers of inflammation and endothelial dysfunction. Therefore, these interpretations should be considered biologically plausible and hypothesis‐generating rather than causal. Another limitation is our reliance on echocardiographic classifications (systolic and diastolic dysfunction) rather than established clinical heart failure phenotypes (HFrEF, HFmrEF, and HFpEF). Diagnosing these clinical syndromes requires concurrent symptoms and biomarkers, which were not uniformly available alongside imaging in our retrospective database. Consequently, our conclusions highlight a pathophysiological vulnerability to impaired myocardial mechanics rather than overt clinical syndromes. Therefore, these findings should be carefully contextualized when compared to clinical trials defined strictly by HFrEF or HFpEF criteria. Lastly, because the cohort derives from a localized healthcare network in southern Taiwan, confirming these results will require prospective tracking across multiple independent centers.

These results hold distinct clinical relevance due to the scarcity of literature analyzing how retinal disease staging relates to incident heart failure. Applying a large sample and standardized ocular imaging, this analysis supports the implementation of parallel screening protocols for both organ systems. Specifically, the identification of any degree of diabetic retinopathy may identify patients who warrant closer cardiovascular assessment. We recommend baseline evaluations (e.g., electrocardiography and natriuretic peptide testing) for patients with mild nonproliferative retinopathy. For those with moderate‐to‐severe or proliferative retinopathy, more comprehensive assessments, such as routine echocardiography, and the proactive optimization of cardioprotective therapies are highly warranted. In the reverse direction, subjects with preexisting heart failure—specifically those showing impaired systolic output—face a greater vulnerability to severe ocular microvascular damage, creating a strong clinical rationale for routine retinal imaging. Subsequent investigations must clarify the exact physiological pathways, including widespread microcirculatory impairment, connecting diabetic eye complications and cardiac dysfunction.

To summarize, advancing stages of diabetic retinal disease function as independent prognostic markers for subsequent heart failure in the type 2 diabetic population. From a bidirectional perspective, preexisting clinical heart failure—especially among individuals with poor renal function or those prescribed diuretic agents—alongside diminished left ventricular systolic and diastolic function, reliably indicates a high probability of advanced ocular damage, regardless of underlying coronary artery disease. Consequently, incorporating retinal evaluations into standard cardiac risk assessments offers a practical strategy to improve early detection and therapeutic management for diabetic heart disease.

## Funding

This work was supported by the Ministry of Science and Technology, Taiwan (MOST‐109‐2314‐B‐037‐088, NSTC‐113‐2314‐B‐037‐097‐MY3), Kaohsiung Medical University Chung‐Ho Memorial Hospital (KMUH111‐1M09, KMUH112‐2M08, KMUH113‐3R19, S11209, S11305, KMUH‐DK(B)110003‐1), and Kaohsiung Medical University (NPUST‐KMU‐111‐P001, NHRIKMU‐111‐I001‐3, KT112P012, KT113P006, NSYSUKMU‐115‐I02).

## Disclosure

The authors have nothing to report.

## Conflicts of Interest

The authors declare no conflicts of interest.

## Supporting information


**Figure S1:** Study flow diagram. This flow diagram illustrates the selection of the study population and analytic cohorts used to evaluate the associations among diabetic retinopathy severity, left ventricular function, and incident heart failure. Adults with type 2 diabetes mellitus who underwent color fundus photography were identified. Participants were stratified by the presence or absence of baseline heart failure. Cross‐sectional analyses assessed the associations of heart failure history, diuretic use, and left ventricular function with diabetic retinopathy severity. Longitudinal analyses evaluated the risk of incident acute heart failure among participants without prior heart failure according to retinopathy severity. DR, diabetic retinopathy; HF, heart failure; LV, left ventricular.

**Figure S2:** eGFR distribution by retinopathy and heart failure. This stacked bar chart illustrates the distribution of estimated glomerular filtration rate categories across diabetic retinopathy severity, stratified by the presence or absence of heart failure. Within each heart failure stratum, proportions of eGFR categories are shown for participants with no apparent diabetic retinopathy, mild nonproliferative diabetic retinopathy, and moderate to severe nonproliferative or proliferative diabetic retinopathy. Patients with heart failure and more advanced retinopathy demonstrated a higher proportion of advanced eGFR categories, highlighting the coexistence of retinal microvascular disease, cardiac dysfunction, and impaired kidney function. eGFR categories were defined as G1 (≥ 90), G2 (60–89), G3 (30–59), G4 (15–29), and G5 (< 15) mL/min/1.73 m^2^. eGFR, estimated glomerular filtration rate.

**Figure S3:** Risk of having moderate and severe diabetic retinopathy in patients with heart failure comorbidities stratified by baseline patient characteristics. This forest plot presents adjusted odds ratios for moderate to severe diabetic retinopathy among patients with heart failure, stratified by baseline demographic and clinical characteristics. Estimates were derived from multivariable ordinal logistic regression models adjusted for age, sex, systolic blood pressure, body mass index, comorbidities, medications, and laboratory variables. Subgroups include age, sex, eGFR categories, hypertension, hyperlipidemia, and statin use. Points represent adjusted odds ratios, and horizontal bars indicate 95% confidence intervals. The vertical reference line denotes an odds ratio of 1.0. eGFR categories were defined as G1 (≥ 90), G2 (60–89), G3 (30–59), G4 (15–29), and G5 (< 15) mL/min/1.73 m^2^. An asterisk indicates statistical significance at *p* < 0.05. The data are presented in odds ratio and 95% confidence interval. eGFR, estimated glomerular filtration rate.

**Figure S4:** Retinopathy severity by heart failure phenotype. This stacked bar chart illustrates the distribution of diabetic retinopathy severity across heart failure phenotypes defined by left ventricular ejection fraction. Participants were categorized as heart failure with normal ejection fraction, heart failure with preserved ejection fraction, and heart failure with reduced or mildly reduced ejection fraction. Within each phenotype, proportions of no apparent diabetic retinopathy, mild nonproliferative diabetic retinopathy, and moderate to severe nonproliferative or proliferative diabetic retinopathy are displayed. More advanced retinopathy severity was observed among patients with reduced or mildly reduced ejection fraction, suggesting an association between retinal microvascular disease and impaired systolic cardiac function.

**Figure S5:** Incident heart failure by retinopathy severity. This Kaplan–Meier curve illustrates the cumulative incidence of incident heart failure among patients with type 2 diabetes mellitus without a prior history of heart failure, stratified by diabetic retinopathy severity at baseline. Participants were categorized as having no apparent diabetic retinopathy, mild nonproliferative diabetic retinopathy, or moderate to severe nonproliferative or proliferative diabetic retinopathy. Increasing retinopathy severity was associated with a progressively higher risk of incident heart failure. Differences among groups were statistically significant by log‐rank testing (*p* < 0.0001).

**Figure S6:** Subgroup analyses of incident heart failure by retinopathy severity. These Kaplan–Meier curves present subgroup analyses evaluating the association between diabetic retinopathy severity and incident heart failure among patients with type 2 diabetes mellitus without a prior history of heart failure. Participants were stratified by baseline characteristics, including age ≥ 65 years (A) and < 65 years (B), sex [male (C), female (D)], eGFR categories G1–G3 (E) and G4–G5 (F), hypertension status [with (G), without (H)], hyperlipidemia status [with (I), without (J)], and statin use [users (K), nonusers (L)]. Across most subgroups, increasing retinopathy severity was consistently associated with a higher risk of incident heart failure. The association was attenuated in patients with advanced kidney disease (eGFR G4–G5) and in those without hypertension. Statistical significance was assessed using log‐rank tests within each subgroup. eGFR categories are defined as G1 (≥ 90), G2 (60–89), G3 (30–59), G4 (15–29), and G5 (< 15) mL/min/1.73 m^2^. eGFR, estimated glomerular filtration rate.

**Figure S7:** Subgroup analysis of the association between diabetic retinopathy categories and the risk of incident heart failure. Diabetic retinopathy Group 1 served as the reference group for all comparisons. The green dots and error bars represent the adjusted hazard ratios and 95% confidence intervals for diabetic retinopathy Group 2 compared to Group 1. The blue dots and error bars represent the adjusted hazard ratios and 95% confidence intervals for diabetic retinopathy Group 3 compared to Group 1. All models were adjusted for age, sex, systolic blood pressure, body mass index, comorbidities, medications, and laboratory variables. The P for interaction was calculated using the likelihood ratio test to assess the homogeneity of the diabetic retinopathy effect across different subgroups.

**Table S1:** Baseline characteristics stratified by left ventricular function.

**Table S2:** Baseline characteristics stratified by echocardiography availability.

**Table S3:** Association between diuretic use and diabetic retinopathy severity in patients with heart failure (*n* = 742).

**Table S4:** Association between heart failure and diabetic retinopathy severity without a history of intravitreal injection of vascular endothelial growth factor inhibitors (*n* = 21 729).

**Table S5:** Association between heart failure and diabetic retinopathy severity by estimated glomerular filtration rate category.

**Table S6:** Incident heart failure according to 5‐group diabetic retinopathy severity (*n* = 21 031).

**Table S7:** Incident heart failure according to diabetic retinopathy severity stratified by insulin use.

**Table S8:** Mediation analysis of renal factors in the association between diabetic retinopathy severity and heart failure.

## Data Availability

Because institutional privacy rules govern the patient‐level electronic medical records within the KMUHRD, the raw datasets used here are not available in public repositories. However, researchers submitting a formal, reasonable request to the corresponding author may gain access to this information, provided they secure formal permission from Kaohsiung Medical University Hospital.

## References

[1] S. Verma and J. J. V. McMurray , “SGLT2 Inhibitors and Mechanisms of Cardiovascular Benefit,” New England Journal of Medicine 382 (2020): 1629–1637.32320570

[2] J. Xie , M. K. Ikram , M. F. Cotch , et al., “Association of Diabetic Macular Edema and Proliferative Diabetic Retinopathy With Cardiovascular Disease: A Systematic Review and Meta‐Analysis,” JAMA Ophthalmology 135 (2017): 586–593. 28472362 10.1001/jamaophthalmol.2017.0988 PMC5593137

[3] N. Cheung , J. J. Wang , S. L. Rogers , et al., “Diabetic Retinopathy and Risk of Heart Failure,” Journal of the American College of Cardiology 51 (2008): 1573–1578. 18420100 10.1016/j.jacc.2007.11.076

[4] Z. Zhen , Y. Chen , K. Shih , et al., “Altered Myocardial Response in Patients With Diabetic Retinopathy: An Exercise Echocardiography Study,” Cardiovascular Diabetology 14 (2015): 123. 26382215 10.1186/s12933-015-0281-5 PMC4574544

[5] D. Aguilar , D. M. Hallman , L. B. Piller , et al., “Adverse Association Between Diabetic Retinopathy and Cardiac Structure and Function,” American Heart Journal 157 (2009): 563–568. 19249430 10.1016/j.ahj.2008.10.019 PMC2830610

[6] Y. Chen , M. Li , Y. Wang , et al., “Association Between Severity of Diabetic Retinopathy and Cardiac Function in Patients With Type 2 Diabetes,” Journal of Diabetes Research 2023 (2023): 6588932. 37323224 10.1155/2023/6588932 PMC10266918

[7] R. M. Lang , L. P. Badano , V. Mor‐Avi , et al., “Recommendations for Cardiac Chamber Quantification by Echocardiography in Adults: An Update From the American Society of Echocardiography and the European Association of Cardiovascular Imaging,” Journal of the American Society of Echocardiography 28 (2015): 1–39.e14. 25559473 10.1016/j.echo.2014.10.003

[8] S. F. Nagueh , O. A. Smiseth , C. P. Appleton , et al., “Recommendations for the Evaluation of Left Ventricular Diastolic Function by Echocardiography: An Update From the American Society of Echocardiography and the European Association of Cardiovascular Imaging,” Journal of the American Society of Echocardiography 29 (2016): 277–314. 27037982 10.1016/j.echo.2016.01.011

[9] S. T. Chiou , H. D. Lin , N. C. Yu , et al., “An Initial Assessment of the Feasibility and Effectiveness of Implementing Diabetes Shared Care System in Taiwan—Some Experiences From I‐Lan County,” Diabetes Research and Clinical Practice 54, no. Suppl 1 (2001): S67–S73. 11580971 10.1016/s0168-8227(01)00311-4

[10] U. Schmidt‐Erfurth , T. Wrba , G. Duftschmid , et al., “Ehealth 2015 Special Issue: Impact of Electronic Health Records on the Completeness of Clinical Documentation Generated During Diabetic Retinopathy Consultations,” Applied Clinical Informatics 06 (2017): 478–487. 10.4338/ACI-2015-03-RA-0028 PMC4586337 26448793

[11] L. A. Inker , N. D. Eneanya , J. Coresh , et al., “New Creatinine‐ and Cystatin C‐Based Equations to Estimate GFR Without Race,” New England Journal of Medicine 385 (2021): 1737–1749. 34554658 10.1056/NEJMoa2102953 PMC8822996

[12] T. T. Chen , K. P. Chung , I. C. Lin , and M. S. Lai , “The Unintended Consequence of Diabetes Mellitus Pay‐for‐Performance (P4P) Program in Taiwan: Are Patients With More Comorbidities or More Severe Conditions Likely to Be Excluded From the P4P Program?,” Health Services Research 46 (2011): 47–60. 20880044 10.1111/j.1475-6773.2010.01182.x PMC3034261

[13] W. Yu , B. Yang , S. Xu , Y. Gao , Y. Huang , and Z. Wang , “Diabetic Retinopathy and Cardiovascular Disease: A Literature Review,” Diabetes, Metabolic Syndrome and Obesity: Targets and Therapy 16 (2023): 4247–4261. 38164419 10.2147/DMSO.S438111 PMC10758178

[14] Y. Li , Y. Liu , S. Liu , et al., “Diabetic Vascular Diseases: Molecular Mechanisms and Therapeutic Strategies,” Signal Transduction and Targeted Therapy 8 (2023): 152. 37037849 10.1038/s41392-023-01400-z PMC10086073

[15] T. Almdal , H. Scharling , J. S. Jensen , and H. Vestergaard , “The Independent Effect of Type 2 Diabetes Mellitus on Ischemic Heart Disease, Stroke, and Death: A Population‐Based Study of 13,000 Men and Women With 20 Years of Follow‐Up,” Archives of Internal Medicine 164 (2004): 1422–1426. 15249351 10.1001/archinte.164.13.1422

[16] T. H. Marwick , R. Ritchie , J. E. Shaw , and D. Kaye , “Implications of Underlying Mechanisms for the Recognition and Management of Diabetic Cardiomyopathy,” Journal of the American College of Cardiology 71 (2018): 339–351. 29348027 10.1016/j.jacc.2017.11.019

[17] S. M. Dunlay , M. M. Givertz , D. Aguilar , et al., “Type 2 Diabetes Mellitus and Heart Failure: A Scientific Statement From the American Heart Association and the Heart Failure Society of America,” Circulation 140 (2019): e294–e324.31167558

[18] W. B. Kannel and D. L. McGee , “Diabetes and Cardiovascular Disease: The Framingham Study,” Journal of the American Medical Association 241 (1979): 2035–2038. 430798 10.1001/jama.241.19.2035

[19] G. Jia , M. A. Hill , and J. R. Sowers , “Diabetic Cardiomyopathy: An Update of Mechanisms Contributing to this Clinical Entity,” Circulation Research 122 (2018): 624–638. 29449364 10.1161/CIRCRESAHA.117.311586 PMC5819359

[20] A. Goldin , J. A. Beckman , A. M. Schmidt , and M. A. Creager , “Advanced Glycation End Products: Sparking the Development of Diabetic Vascular Injury,” Circulation 114 (2006): 597–605. 16894049 10.1161/CIRCULATIONAHA.106.621854

[21] V. R. Taqueti and M. F. Di Carli , “Coronary Microvascular Disease Pathogenic Mechanisms and Therapeutic Options: JACC State‐of‐the‐Art Review,” Journal of the American College of Cardiology 72 (2018): 2625–2641. 30466521 10.1016/j.jacc.2018.09.042 PMC6296779

[22] M. V. van Hecke , J. M. Dekker , C. D. Stehouwer , et al., “Diabetic Retinopathy is Associated With Mortality and Cardiovascular Disease Incidence: The EURODIAB Prospective Complications Study,” Diabetes Care 28 (2005): 1383–1389. 15920056 10.2337/diacare.28.6.1383

[23] S. S. Soedamah‐Muthu , N. Chaturvedi , M. Toeller , et al., “Risk Factors for Coronary Heart Disease in Type 1 Diabetic Patients in Europe: The EURODIAB Prospective Complications Study,” Diabetes Care 27 (2004): 530–537. 14747240 10.2337/diacare.27.2.530

[24] J. J. Drinkwater , T. M. E. Davis , V. Hellbusch , A. W. Turner , D. G. Bruce , and W. A. Davis , “Retinopathy Predicts Stroke but Not Myocardial Infarction in Type 2 Diabetes: The Fremantle Diabetes Study Phase II,” Cardiovascular Diabetology 19 (2020): 43. 32234054 10.1186/s12933-020-01018-3 PMC7110810

[25] R. Klein , A. R. Sharrett , B. E. K. Klein , et al., “Are Retinal Arteriolar Abnormalities Related to Atherosclerosis?,” Arteriosclerosis, Thrombosis, and Vascular Biology 20 (2000): 1644–1650. 10845884 10.1161/01.atv.20.6.1644

[26] T. Y. Wong , W. Rosamond , P. P. Chang , et al., “Retinopathy and Risk of Congestive Heart Failure,” Journal of the American Medical Association 293 (2005): 63–69. 15632337 10.1001/jama.293.1.63

[27] R. Hiller , R. D. Sperduto , M. J. Podgor , F. L. Ferris, 3rd , and P. W. Wilson , “Diabetic Retinopathy and Cardiovascular Disease in Type II Diabetics. The Framingham Heart Study and the Framingham Eye Study,” American Journal of Epidemiology 128 (1988): 402–409. 3293436 10.1093/oxfordjournals.aje.a114980

[28] A. Palazzuoli , G. Ruocco , C. Ronco , and P. A. McCullough , “Loop Diuretics in Acute Heart Failure: Beyond the Decongestive Relief for the Kidney,” Critical Care 19 (2015): 296. 26335137 10.1186/s13054-015-1017-3 PMC4559070

[29] H. A. Cooper , D. L. Dries , C. E. Davis , Y. L. Shen , and M. J. Domanski , “Diuretics and Risk of Arrhythmic Death in Patients With Left Ventricular Dysfunction,” Circulation 100 (1999): 1311–1315. 10491376 10.1161/01.cir.100.12.1311

[30] R. Deva , M. A. Alias , D. Colville , et al., “Vision‐Threatening Retinal Abnormalities in Chronic Kidney Disease Stages 3 to 5,” Clinical Journal of the American Society of Nephrology 6 (2011): 1866–1871. 21784818 10.2215/CJN.10321110 PMC3359545

[31] W. J. Lee , L. Sobrin , M. J. Lee , M. H. Kang , M. Seong , and H. Cho , “The Relationship Between Diabetic Retinopathy and Diabetic Nephropathy in a Population‐Based Study in Korea (KNHANES V‐2, 3),” Investigative Ophthalmology & Visual Science 55 (2014): 6547–6553. 25205863 10.1167/iovs.14-15001

[32] T. E. Farrah , D. Pugh , F. A. Chapman , et al., “Choroidal and Retinal Thinning in Chronic Kidney Disease Independently Associate With eGFR Decline and Are Modifiable With Treatment,” Nature Communications 14 (2023): 7720. 10.1038/s41467-023-43125-1 PMC10697963 38052813

[33] M. Yamanouchi , M. Mori , J. Hoshino , et al., “Retinopathy Progression and the Risk of End‐Stage Kidney Disease: Results From a Longitudinal Japanese Cohort of 232 Patients With Type 2 Diabetes and Biopsy‐Proven Diabetic Kidney Disease,” BMJ Open Diabetes Research & Care 7 (2019): e000726. 10.1136/bmjdrc-2019-000726 PMC6861100 31798893

[34] P. M. Seferović and W. J. Paulus , “Clinical Diabetic Cardiomyopathy: A Two‐Faced Disease With Restrictive and Dilated Phenotypes,” European Heart Journal 36 (2015): 1718–1727. 25888006 10.1093/eurheartj/ehv134

[35] J. Weerts , B. L. M. Schroen , A. Barandiarán Aizpurua , et al., “Microvascular Dysfunction Across Organs in Heart Failure With Preserved Ejection Fraction: The PROSE‐HFpEF Case‐Control Study,” Cardiovascular Diabetology 24 (2025): 310. 40739496 10.1186/s12933-025-02850-1 PMC12312529

