# Supplementary figures and images for: Diabetic Retinopathy Severity and Heart Failure Outcomes in Type 2 Diabetes Mellitus
Source: J Diabetes. 2026 Jul 2;18(7):e70235. doi: 10.1111/1753-0407.70235 (PMC13328843; doi:10.1111/1753-0407.70235)

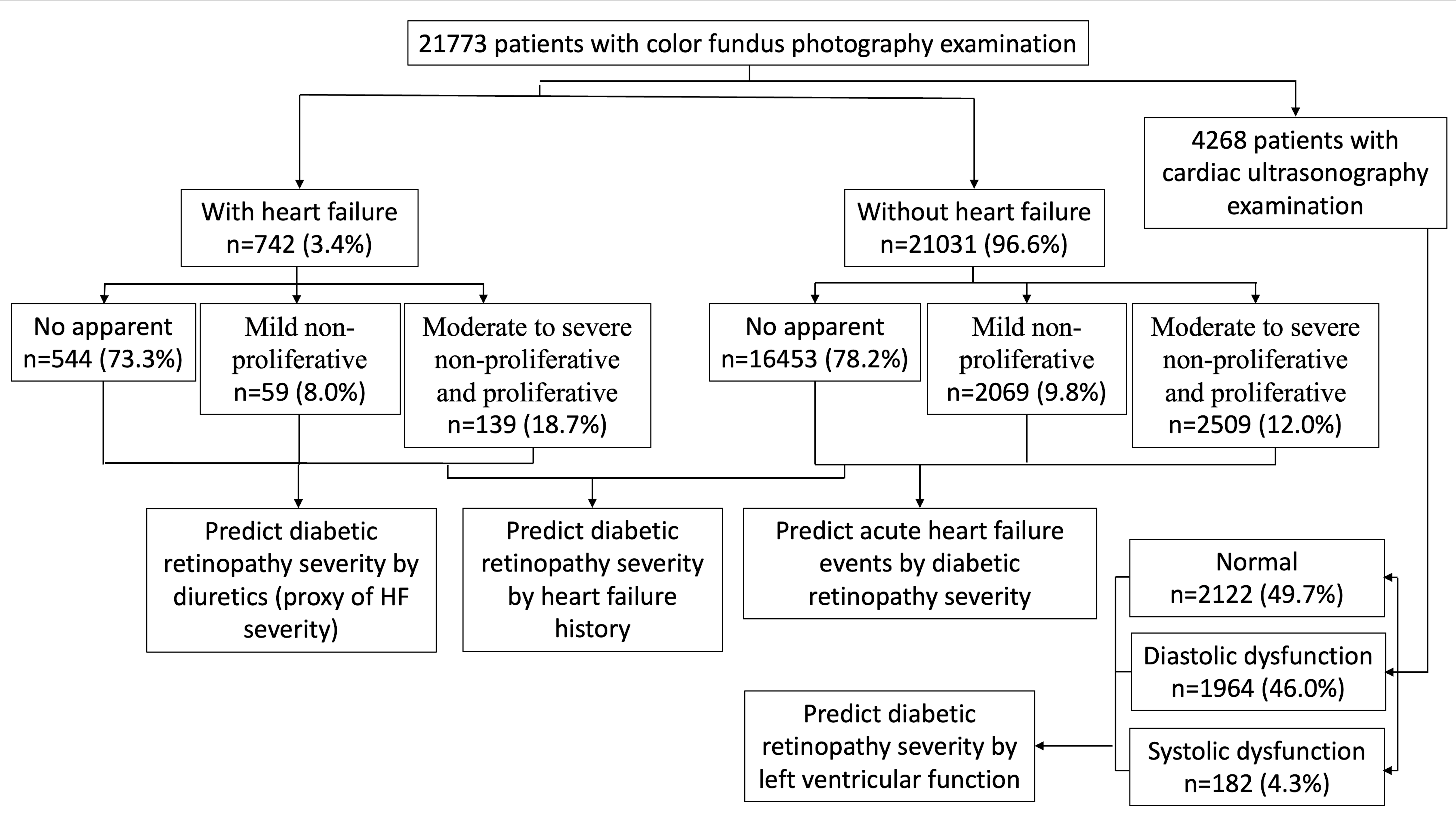

Supplement: Supplementary file 1 — Figure S1: Study flow diagram. This flow diagram illustrates the selection of the study population and analytic cohorts used to evaluate the associations among diabetic retinopathy severity, left ventricular function, and incident heart failure. Adults with type 2 diabetes mellitus who underwent color fundus photography were identified. Participants were stratified by the presence or absence of baseline heart failure. Cross‐sectional analyses assessed the associations of heart failure history, diuretic use, and left ventricular function with diabetic retinopathy severity. Longitudinal analyses evaluated the risk of incident acute heart failure among participants without prior heart failure according to retinopathy severity. DR, diabetic retinopathy; HF, heart failure; LV, left ventricular. [file JDB-18-e70235-s002.png]

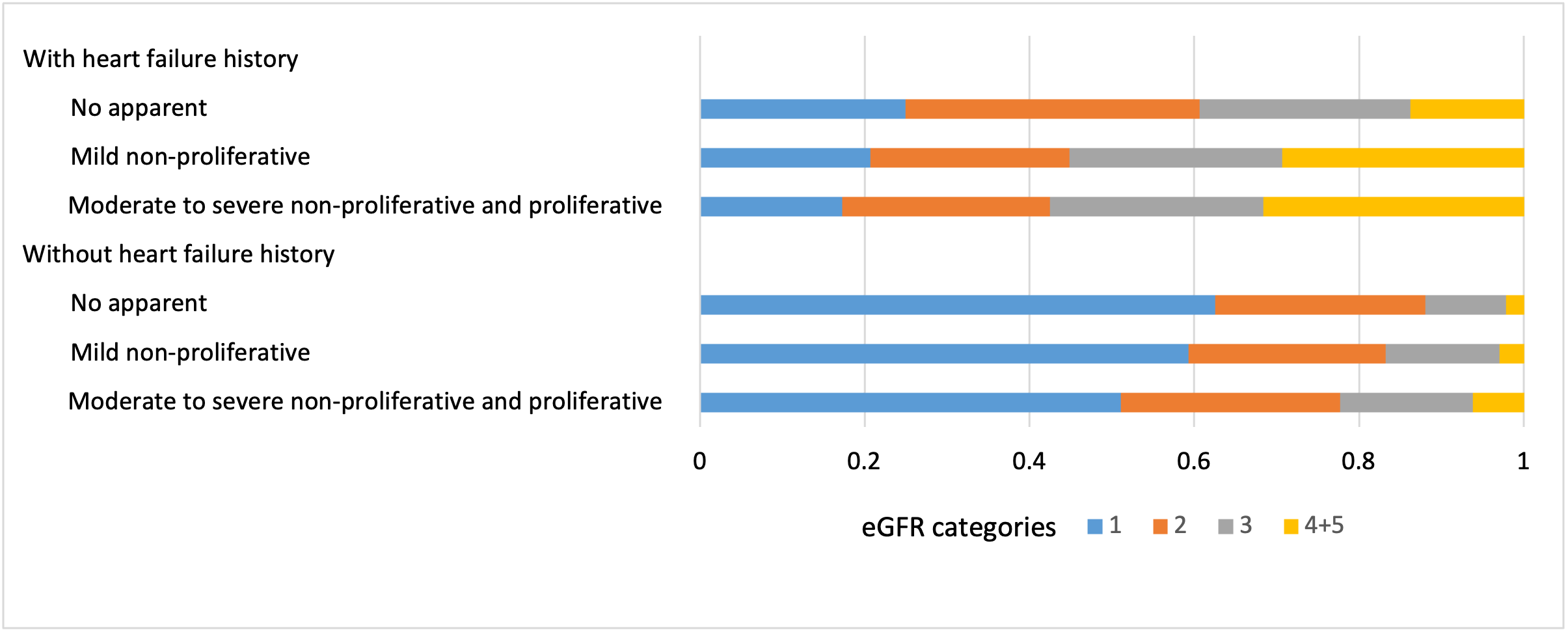

Supplement: Supplementary file 2 — Figure S2: eGFR distribution by retinopathy and heart failure. This stacked bar chart illustrates the distribution of estimated glomerular filtration rate categories across diabetic retinopathy severity, stratified by the presence or absence of heart failure. Within each heart failure stratum, proportions of eGFR categories are shown for participants with no apparent diabetic retinopathy, mild nonproliferative diabetic retinopathy, and moderate to severe nonproliferative or proliferative diabetic retinopathy. Patients with heart failure and more advanced retinopathy demonstrated a higher proportion of advanced eGFR categories, highlighting the coexistence of retinal microvascular disease, cardiac dysfunction, and impaired kidney function. eGFR categories were defined as G1 (≥ 90), G2 (60–89), G3 (30–59), G4 (15–29), and G5 (< 15) mL/min/1.73 m2. eGFR, estimated glomerular filtration rate. [file JDB-18-e70235-s006.png]

## Adjusted odds ratio for diabetic retinopathy severity by congestive heart failure status

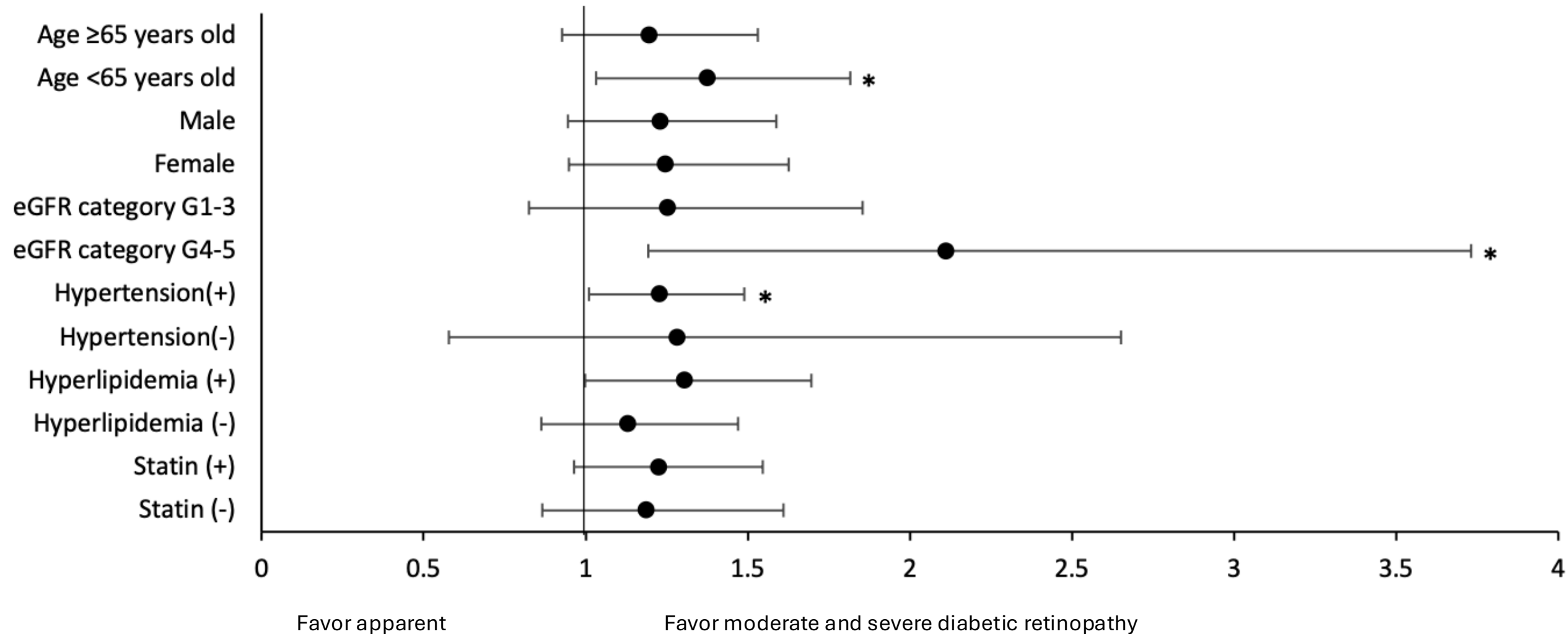

Supplement: Supplementary file 3 — Figure S3: Risk of having moderate and severe diabetic retinopathy in patients with heart failure comorbidities stratified by baseline patient characteristics. This forest plot presents adjusted odds ratios for moderate to severe diabetic retinopathy among patients with heart failure, stratified by baseline demographic and clinical characteristics. Estimates were derived from multivariable ordinal logistic regression models adjusted for age, sex, systolic blood pressure, body mass index, comorbidities, medications, and laboratory variables. Subgroups include age, sex, eGFR categories, hypertension, hyperlipidemia, and statin use. Points represent adjusted odds ratios, and horizontal bars indicate 95% confidence intervals. The vertical reference line denotes an odds ratio of 1.0. eGFR categories were defined as G1 (≥ 90), G2 (60–89), G3 (30–59), G4 (15–29), and G5 (< 15) mL/min/1.73 m2. An asterisk indicates statistical significance at p < 0.05. The data are presented in odds ratio and 95% confidence interval. eGFR, estimated glomerular filtration rate. [file JDB-18-e70235-s003.pdf]

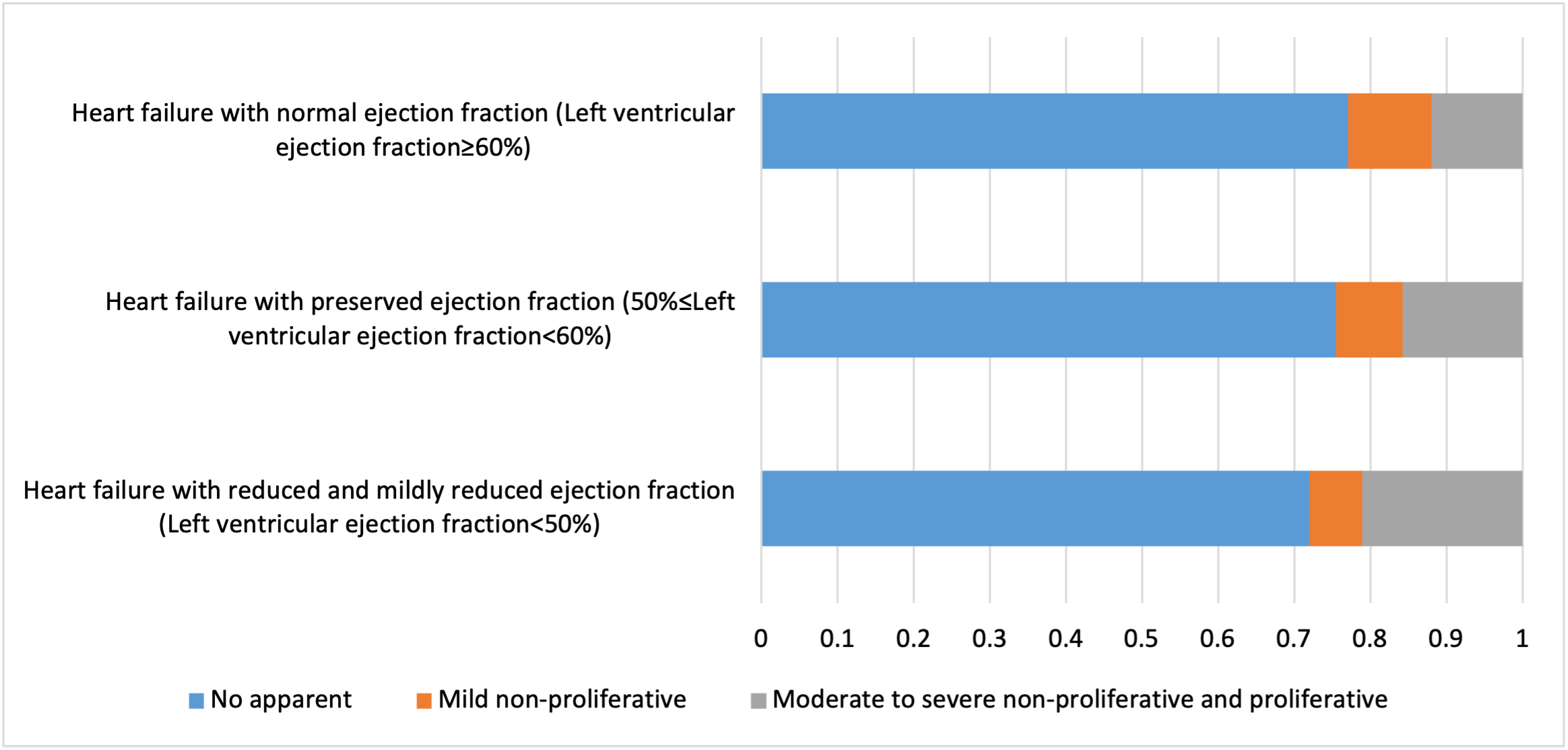

Supplement: Supplementary file 4 — Figure S4: Retinopathy severity by heart failure phenotype. This stacked bar chart illustrates the distribution of diabetic retinopathy severity across heart failure phenotypes defined by left ventricular ejection fraction. Participants were categorized as heart failure with normal ejection fraction, heart failure with preserved ejection fraction, and heart failure with reduced or mildly reduced ejection fraction. Within each phenotype, proportions of no apparent diabetic retinopathy, mild nonproliferative diabetic retinopathy, and moderate to severe nonproliferative or proliferative diabetic retinopathy are displayed. More advanced retinopathy severity was observed among patients with reduced or mildly reduced ejection fraction, suggesting an association between retinal microvascular disease and impaired systolic cardiac function. [file JDB-18-e70235-s008.png]

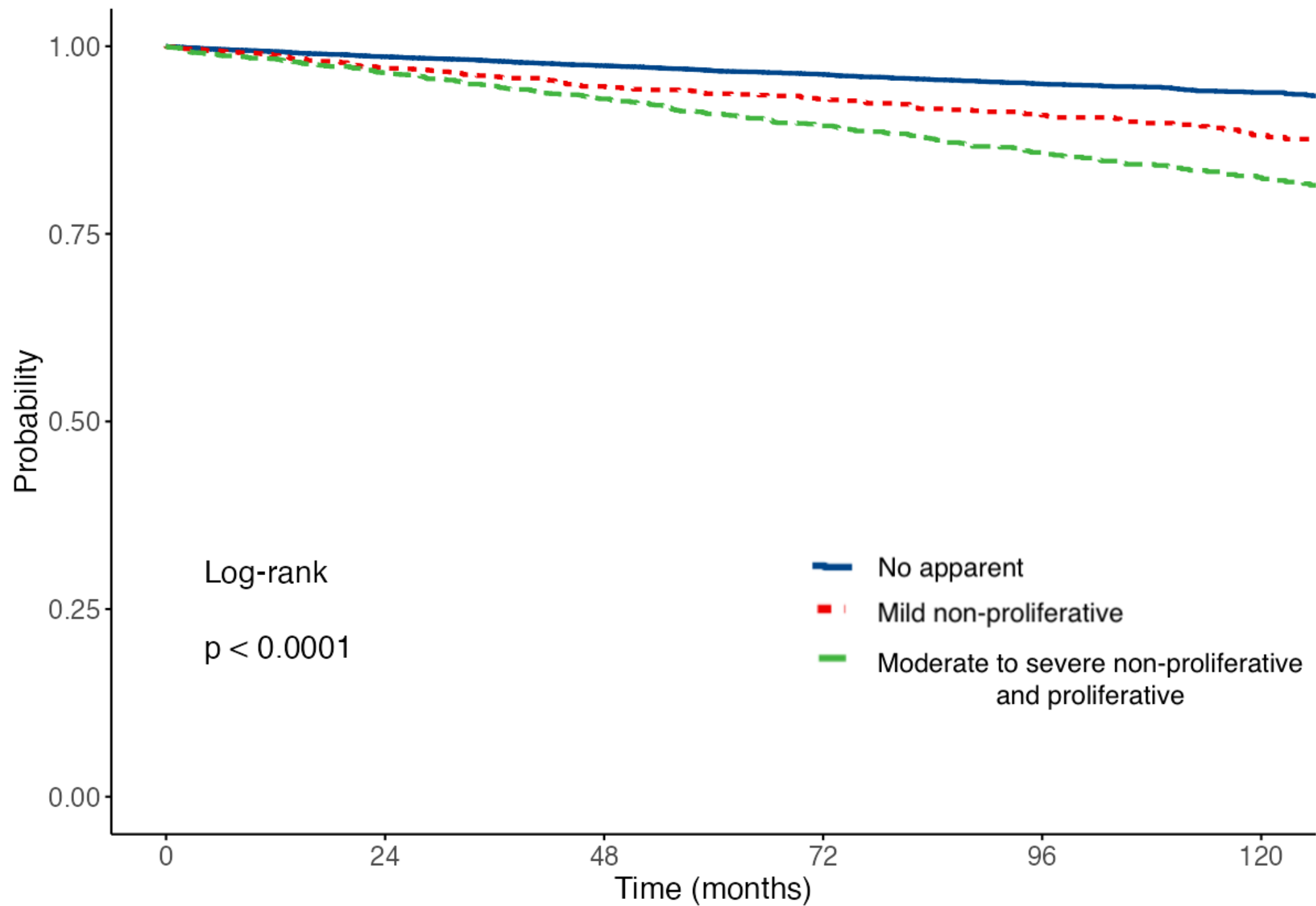

Supplement: Supplementary file 5 — Figure S5: Incident heart failure by retinopathy severity. This Kaplan–Meier curve illustrates the cumulative incidence of incident heart failure among patients with type 2 diabetes mellitus without a prior history of heart failure, stratified by diabetic retinopathy severity at baseline. Participants were categorized as having no apparent diabetic retinopathy, mild nonproliferative diabetic retinopathy, or moderate to severe nonproliferative or proliferative diabetic retinopathy. Increasing retinopathy severity was associated with a progressively higher risk of incident heart failure. Differences among groups were statistically significant by log‐rank testing (p < 0.0001). [file JDB-18-e70235-s010.pdf]

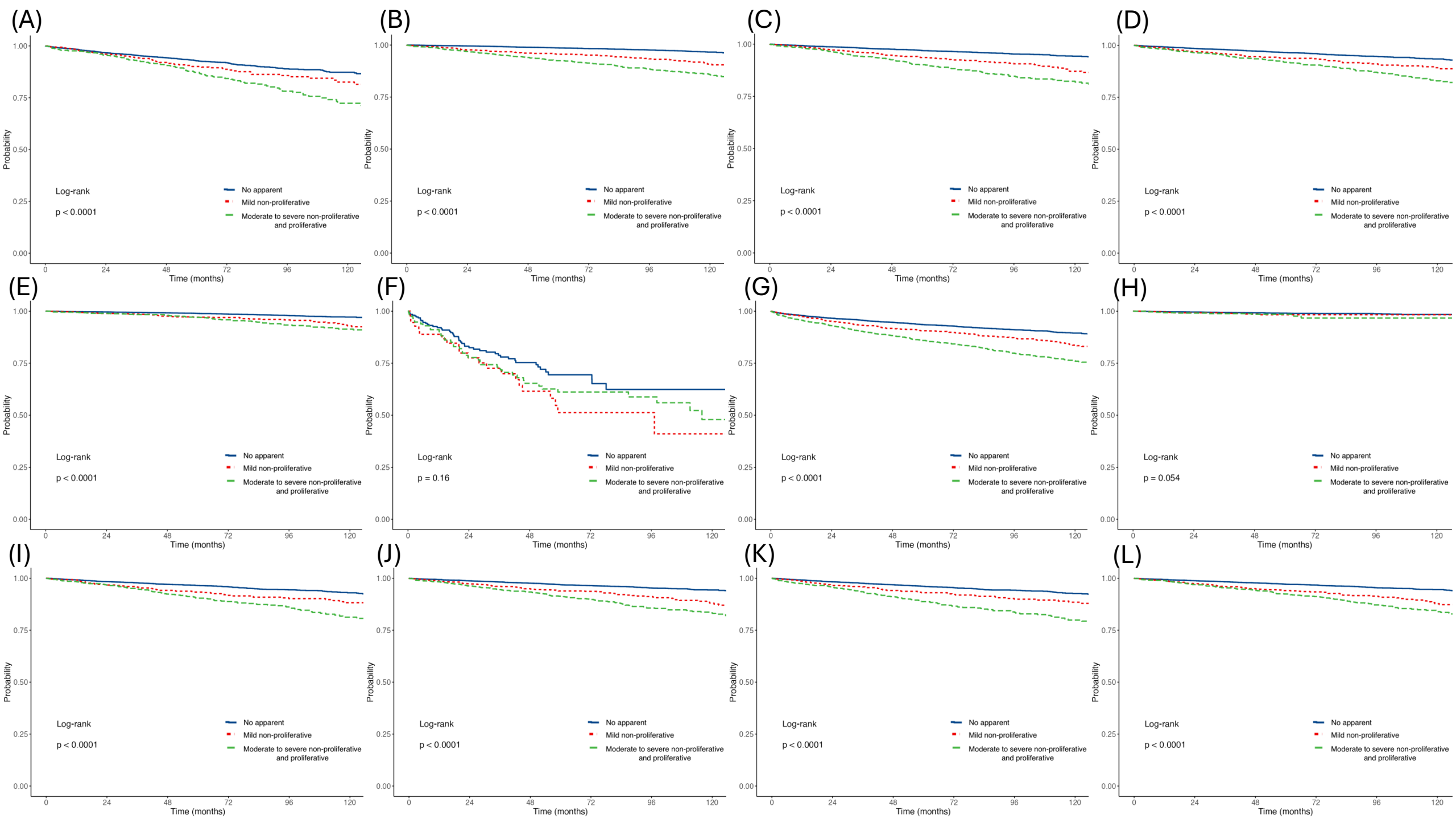

Supplement: Supplementary file 6 — Figure S6: Subgroup analyses of incident heart failure by retinopathy severity. These Kaplan–Meier curves present subgroup analyses evaluating the association between diabetic retinopathy severity and incident heart failure among patients with type 2 diabetes mellitus without a prior history of heart failure. Participants were stratified by baseline characteristics, including age ≥ 65 years (A) and < 65 years (B), sex [male (C), female (D)], eGFR categories G1–G3 (E) and G4–G5 (F), hypertension status [with (G), without (H)], hyperlipidemia status [with (I), without (J)], and statin use [users (K), nonusers (L)]. Across most subgroups, increasing retinopathy severity was consistently associated with a higher risk of incident heart failure. The association was attenuated in patients with advanced kidney disease (eGFR G4–G5) and in those without hypertension. Statistical significance was assessed using log‐rank tests within each subgroup. eGFR categories are defined as G1 (≥ 90), G2 (60–89), G3 (30–59), G4 (15–29), and G5 (< 15) mL/min/1.73 m2. eGFR, estimated glomerular filtration rate. [file JDB-18-e70235-s009.pdf]

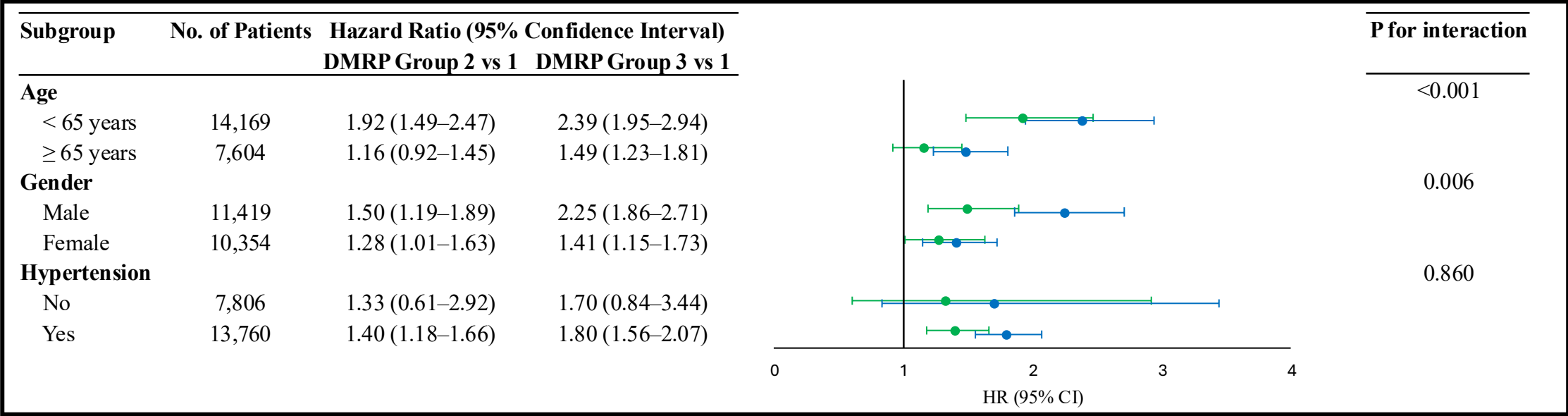

Supplement: Supplementary file 7 — Figure S7: Subgroup analysis of the association between diabetic retinopathy categories and the risk of incident heart failure. Diabetic retinopathy Group 1 served as the reference group for all comparisons. The green dots and error bars represent the adjusted hazard ratios and 95% confidence intervals for diabetic retinopathy Group 2 compared to Group 1. The blue dots and error bars represent the adjusted hazard ratios and 95% confidence intervals for diabetic retinopathy Group 3 compared to Group 1. All models were adjusted for age, sex, systolic blood pressure, body mass index, comorbidities, medications, and laboratory variables. The P for interaction was calculated using the likelihood ratio test to assess the homogeneity of the diabetic retinopathy effect across different subgroups. [file JDB-18-e70235-s015.pdf]
