# Supplementary material for: Diabetic Retinopathy Severity and Heart Failure Outcomes in Type 2 Diabetes Mellitus
Source: J Diabetes. 2026 Jul 2;18(7):e70235. doi: 10.1111/1753-0407.70235 (PMC13328843; doi:10.1111/1753-0407.70235)
Supplement: Supplementary file 8 — Table S1: Baseline characteristics stratified by left ventricular function. [file JDB-18-e70235-s014.docx]

**Supplementary Table 1.** Baseline characteristics stratified by left ventricular function

|  | Normal (N=2,122) | Diastolic dysfunction (N=1,964) | Systolic dysfunction (N=182) | *p-*value |
| --- | --- | --- | --- | --- |
| **Baseline demographic** |  |  |  |  |
| Age (years) | 62.7 ± 13.0 | 66.6 ± 11.3 | 66.2 ± 11.3 | < 0.001 |
| Male Gender, n (%) | 1180 (55.6) | 957 (48.7) | 130 (71.4) | < 0.001 |
| Systolic blood pressure (mmHg) | 133.3 ± 14.7 | 135.8 ± 14.6 | 132.6 ± 19.0 | < 0.001 |
| Body mass index (kg/m^2^) | 26.5 ± 4.7 | 26.4 ± 4.5 | 26.5 ± 4.5 | 0.526 |
| **Comorbidities (n, %)** |  |  |  |  |
| eGFR category (mL/min/1.73m²) |  |  |  | < 0.001 |
| G1 (≥ 90) | 927 (43.7) | 770 (39.2) | 44 (24.2) |  |
| G2 (60–89) | 671 (31.6) | 630 (32.1) | 61 (33.5) |  |
| G3 (30–59) | 383 (18.0) | 399 (20.3) | 50 (27.5) |  |
| G4+5 (< 30) | 141 (6.7) | 165 (8.4) | 27 (14.8) |  |
| Hypertension | 1698 (80.0) | 1702 (86.7) | 159 (87.4) | < 0.001 |
| Coronary artery disease | 544 (25.6) | 453 (23.1) | 91 (50.0) | < 0.001 |
| Atrial fibrillation | 171 (8.1) | 50 (2.5) | 27 (14.8) | < 0.001 |
| Hyperlipidemia | 1014 (47.8) | 928 (47.3) | 84 (46.2) | 0.883 |
| Chronic obstructive pulmonary disease | 99 (4.7) | 102 (5.2) | 15 (8.2) | 0.101 |
| **Medications (n, %)** |  |  |  |  |
| Beta blocker | 1002 (47.2) | 811 (41.3) | 120 (65.9) | < 0.001 |
| ACEI/ARB | 1195 (56.3) | 1090 (55.5) | 112 (61.5) | 0.285 |
| Statin | 1081 (50.9) | 948 (48.3) | 113 (62.1) | 0.001 |
| IVI Anti-VEGF | 9 (0.4) | 9 (0.5) | 1 (0.5) | 0.964 |
| SGLT2 inhibitor | 99 (4.7) | 18 (9.9) | 102 (5.2) | 0.009 |
| Glucagon-Like Peptide-1 agonist | 8 (0.4) | 5 (2.7) | 29 (1.5) | <0.001 |
| **Laboratory data (mean ± SD)** |  |  |  |  |
| Low-density lipoprotein (mg/dl) | 96.2 ± 36.8 | 99.7 ± 36.9 | 93.2 ± 38.8 | 0.002 |
| Glycated hemoglobin (%) | 7.9 ± 2.1 | 8.1 ± 2.2 | 8.4 ± 2.2 | < 0.001 |
| eGFR (mL/min/1.73 m^2^) | 79.0 ± 28.4 | 75.0 ± 28.2 | 65.3 ± 28.9 | < 0.001 |
| **Diabetic retinopathy (n, %)** |  |  |  | < 0.001 |
| No apparent | 1696 (79.9) | 1509 (76.8) | 119 (65.4) |  |
| Mild non-proliferative diabetic retinopathy | 123 (5.8) | 166 (8.5) | 15 (8.2) |  |
| Moderate to severe non-proliferative diabetic retinopathy and proliferative diabetic retinopathy | 303 (14.3) | 289 (14.7) | 48 (26.4) |  |

The data are expressed as mean ± standard deviation unless otherwise stated.

eGFR is calculated by the CKD-EPI (Chronic Kidney Disease Epidemiology Collaboration) equation

Abbreviations: ACEI = angiotensin-converting enzyme inhibitor; ARB = angiotensin II receptor blocker; SGLT2 = Sodium-Glucose Cotransporter 2; eGFR = estimated glomerular filtration rate; SD = standard deviation; IVI = intravitreal injection VEGF = vascular endothelial growth factor.
