# Supplementary material for: Diabetic Retinopathy Severity and Heart Failure Outcomes in Type 2 Diabetes Mellitus
Source: J Diabetes. 2026 Jul 2;18(7):e70235. doi: 10.1111/1753-0407.70235 (PMC13328843; doi:10.1111/1753-0407.70235)
Supplement: Supplementary file 9 — Table S2: Baseline characteristics stratified by echocardiography availability. [file JDB-18-e70235-s013.docx]

**Supplementary Table 2.** Baseline characteristics stratified by echocardiography availability

|  | **No cardiac echocardiography (n=17,505)** | **With cardiac echocardiography (n=4,268)** | **p-value** |
| --- | --- | --- | --- |
| **Baseline demographic** |  |  |  |
| Age (years) | 57.9 ± 13.6 | 64.7 ± 12.3 | < 0.001 |
| Male Gender, n (%) | 9152 (52.3) | 2267 (53.1) | 0.328 |
| Systolic blood pressure (mmHg) | 133.5 ± 14.3 | 134.4 ± 14.9 | < 0.001 |
| Body mass index (kg/m^2^) | 26.2 ± 4.5 | 26.4 ± 4.6 | < 0.001 |
| **Comorbidities (n, %)** |  |  |  |
| eGFR category (mL/min/1.73m²) |  |  | < 0.001 |
| G1 (≥ 90) | 11171 (64.1) | 1739 (40.8) |  |
| G2 (60–89) | 4215 (24.2) | 1361 (31.9) |  |
| G3 (30–59) | 1652 (9.5) | 831 (19.5) |  |
| G4+5 (< 30) | 298 (1.7) | 206 (4.8) |  |
| Hypertension | 10408 (59.5) | 3559 (83.4) | < 0.001 |
| Coronary artery disease | 755 (4.3) | 1088 (25.5) | < 0.001 |
| Heart failure | 220 (1.3) | 522 (12.2) | < 0.001 |
| Atrial fibrillation | 104 (0.6) | 248 (5.8) | < 0.001 |
| Hyperlipidemia | 6079 (34.7) | 2026 (47.5) | < 0.001 |
| Chronic obstructive pulmonary disease | 295 (1.7) | 216 (5.1) | < 0.001 |
| **Medications (n, %)** |  |  |  |
| Beta blocker | 2622 (15.0) | 1933 (45.3) | < 0.001 |
| ACEI/ARB | 5695 (32.5) | 2397 (56.2) | < 0.001 |
| Statin | 6173 (35.3) | 2142 (50.2) | < 0.001 |
| IVI Anti-VEGF | 25 (0.1) | 19 (0.4) | < 0.001 |
| SGLT2 inhibitor | 769 (4.4) | 219 (5.1) | 0.038 |
| Glucagon-Like Peptide-1 agonist | 134 (0.8) | 42 (1.0) | 0.153 |
| **Laboratory data** |  |  |  |
| Low-density lipoprotein (mg/dl) | 108.2 ± 38.2 | 97.7 ± 37.0 | < 0.001 |
| Glycated hemoglobin (%) | 8.5 ± 2.4 | 8.0 ± 2.1 | < 0.001 |
| eGFR (mL/min/1.73 m^2^) | 91.9 ± 24.3 | 76.6 ± 28.5 | < 0.001 |

The data are expressed as mean ± standard deviation unless otherwise stated.

eGFR is calculated by the CKD-EPI (Chronic Kidney Disease Epidemiology Collaboration) equation

Abbreviations: ACEI = angiotensin-converting enzyme inhibitor; ARB = angiotensin II receptor blocker; SGLT2 = Sodium-Glucose Cotransporter 2; eGFR = estimated glomerular filtration rate; IVI = intravitreal injection; VEGF = vascular endothelial growth factor.
