# Supplementary material for: Diabetic Retinopathy Severity and Heart Failure Outcomes in Type 2 Diabetes Mellitus
Source: J Diabetes. 2026 Jul 2;18(7):e70235. doi: 10.1111/1753-0407.70235 (PMC13328843; doi:10.1111/1753-0407.70235)
Supplement: Supplementary file 10 — Table S3: Association between diuretic use and diabetic retinopathy severity in patients with heart failure (n = 742). [file JDB-18-e70235-s004.docx]

**Supplementary Table 3**. Association between diuretic use and diabetic retinopathy severity in patients with heart failure (n=742)

| Diabetic retinopathy severity |  |  |
| --- | --- | --- |
|  | Odds ratio (95% Confidence Interval) | *P* value |
| Model 1 | 2.26 (1.57-3.30) | < 0.001 |
| Model 2 | 2.25 (1.55-3.30) | < 0.001 |
| Model 3 | 2.18 (1.49-3.21) | < 0.001 |
| Model 4 | 1.84 (1.24-2.75) | 0.003 |

This sensitivity analysis evaluated diuretic use as a proxy for heart failure severity.

Model 1: Age, gender, systolic blood pressure, body mass index

Model 2: Model 1 + comorbidity (hypertension, coronary artery disease, atrial fibrillation, chronic obstructive pulmonary disease)

Model 3: Model 2 + medications (angiotensin converting enzyme inhibitor/ angiotensin Ⅱ receptor blocker, beta blocker, statin, SGLT2 inhibitors, GLP-1 receptor agonists)

Model 4: Model 3 + laboratory data (low-density lipoprotein-cholesterol, glycated hemoglobin, estimated glomerular filtration rate measured by CKD-EPI (Chronic Kidney Disease Epidemiology Collaboration)

Abbreviations: SGLT2 = Sodium-Glucose Cotransporter 2; GLP-1 = Glucagon-Like Peptide-1.
