# Supplementary material for: Diabetic Retinopathy Severity and Heart Failure Outcomes in Type 2 Diabetes Mellitus
Source: J Diabetes. 2026 Jul 2;18(7):e70235. doi: 10.1111/1753-0407.70235 (PMC13328843; doi:10.1111/1753-0407.70235)
Supplement: Supplementary file 11 — Table S4: Association between heart failure and diabetic retinopathy severity without a history of intravitreal injection of vascular endothelial growth factor inhibitors (n = 21 729). [file JDB-18-e70235-s011.docx]

**Supplementary Table 4**. Association between heart failure and diabetic retinopathy severity without a history of intravitreal injection of vascular endothelial growth factor inhibitors (n=21,729)

| Diabetic retinopathy severity |  |  |
| --- | --- | --- |
|  | Odds ratio (95% Confidence interval) | *P* value |
| Model 1 | 1.49 (1.25-1.77) | < 0.001 |
| Model 2 | 1.46 (1.21-1.75) | < 0.001 |
| Model 3 | 1.45 (1.20-1.74) | < 0.001 |
| Model 4 | 1.22 (1.01-1.47) | 0.040 |

This sensitivity analysis excluded participants with prior intravitreal anti–vascular endothelial growth factor therapy.

Model 1: Age, gender, systolic blood pressure, body mass index

Model 2: Model 1 + comorbidity (hypertension, coronary artery disease, atrial fibrillation, chronic obstructive pulmonary disease)

Model 3: Model 2 + medications (angiotensin converting enzyme inhibitor/ angiotensin Ⅱ receptor blocker, beta blocker, statin, SGLT2 inhibitors, GLP-1 receptor agonists)

Model 4: Model 3 + laboratory data (low-density lipoprotein-cholesterol, glycated hemoglobin, estimated glomerular filtration rate measured by CKD-EPI (Chronic Kidney Disease Epidemiology Collaboration))

Abbreviations: SGLT2 = Sodium-Glucose Cotransporter 2; GLP-1 = Glucagon-Like Peptide-1.
