# Supplementary material for: Diabetic Retinopathy Severity and Heart Failure Outcomes in Type 2 Diabetes Mellitus
Source: J Diabetes. 2026 Jul 2;18(7):e70235. doi: 10.1111/1753-0407.70235 (PMC13328843; doi:10.1111/1753-0407.70235)
Supplement: Supplementary file 12 — Table S5: Association between heart failure and diabetic retinopathy severity by estimated glomerular filtration rate category. [file JDB-18-e70235-s005.docx]

**Supplementary Table 5**. Association between heart failure and diabetic retinopathy severity by estimated glomerular filtration rate category

| Diabetic retinopathy severity |  |  |
| --- | --- | --- |
| eGFR category G1-3 (N= 21,041) |  |  |
|  | Odds ratio (95% Confidence Interval) | *P* value |
| Model 1 | 1.23 (0.82-1.77) | 0.299 |
| Model 2 | 1.25 (0.83-1.84) | 0.266 |
| Model 3 | 1.32 (0.87-1.94) | 0.179 |
| Model 4 | 1.26 (0.83-1.87) | 0.260 |
| eGFR category G4-5 (N=734) |  |  |
|  | Odds ratio (95% Confidence Interval) | *P* value |
| Model 1 | 2.00 (1.18-3.37) | 0.009 |
| Model 2 | 2.07 (1.18-3.63) | 0.011 |
| Model 3 | 2.09 (1.18-3.68) | 0.011 |
| Model 4 | 2.13 (1.20-3.76) | 0.010 |

Model 1: Age, gender, systolic blood pressure, body mass index

Model 2: Model 1 + comorbidity (hypertension, coronary artery disease, atrial fibrillation, chronic obstructive pulmonary disease)

Model 3: Model 2 + medications (angiotensin converting enzyme inhibitor/ angiotensin Ⅱ receptor blocker, beta blocker, statin, SGLT2 inhibitors, GLP-1 receptor agonists)

Model 4: Model 3 + laboratory data (low-density lipoprotein-cholesterol, glycated hemoglobin, estimated glomerular filtration rate measured by CKD-EPI (Chronic Kidney Disease Epidemiology Collaboration))
eGFR categories were defined as G1 (≥90), G2 (60–89), G3 (30–59), G4 (15–29), and G5 (<15) mL/min/1.73 m².

Abbreviations: eGFR= estimated glomerular filtration rate; SGLT2 = Sodium-Glucose Cotransporter 2; GLP-1 = Glucagon-Like Peptide-1.
