# Supplementary material for: Diabetic Retinopathy Severity and Heart Failure Outcomes in Type 2 Diabetes Mellitus
Source: J Diabetes. 2026 Jul 2;18(7):e70235. doi: 10.1111/1753-0407.70235 (PMC13328843; doi:10.1111/1753-0407.70235)
Supplement: Supplementary file 13 — Table S6: Incident heart failure according to 5‐group diabetic retinopathy severity (n = 21 031). [file JDB-18-e70235-s007.docx]

**Supplementary Table 6**. Incident heart failure according to 5-group diabetic retinopathy severity (n=21,031)

|  | No apparent (n=16,453) | Mild non-proliferative (n=2,069) | Moderate non-proliferative (n=1,756) | Severe non-proliferative (n=303) | Proliferative (n=450) | P for trend |
| --- | --- | --- | --- | --- | --- | --- |
| Incident heart failure (n) | 359 | 100 | 100 | 21 | 47 |  |
| Person-year | 55380.7 | 7583.42 | 6351.91 | 1029.64 | 1678.1 |  |
| Incidence rate (per 1,000 person-year) | 6.48 (5.83-7.19) | 13.19 (10.73-16.04) | 15.74 (12.81-19.15) | 20.40 (12.63-31.18) | 28.01 (20.58-37.24) |  |
| Crude Incidence rate ratio | Reference | 2.03 (1.63-2.54) | 2.43 (1.95-3.03) | 3.15 (2.03-4.89) | 4.32 (3.19-5.86) | <0.001 |
| Adjusted Incidence rate ratio | Reference | 1.64 (1.32-2.06) | 1.79 (1.43-2.25) | 2.22 (1.42-3.47) | 1.88 (1.37-2.59) | <0.001 |
| Hazard ratio |  |  |  |  |  |  |
| Model 1 | Reference | 1.91 (1.53-2.38) | 2.38 (1.90-2.98) | 3.67 (2.36-5.71) | 4.58 (3.37-6.23) | <0.001 |
| Model 2 | Reference | 1.90 (1.52-2.37) | 2.32 (1.85-2.90) | 3.57 (2.29-5.56) | 4.04 (2.96-5.50) | <0.001 |
| Model 3 | Reference | 1.88 (1.50-2.35) | 2.29 (1.83-2.87) | 3.52 (2.26-5.50) | 3.92 (2.87-5.34) | <0.001 |
| Model 4 | Reference | 1.64 (1.31-2.05) | 1.79 (1.43-2.25) | 2.20 (1.41-3.44) | 1.87 (1.36-2.58) | <0.001 |

The data are expressed as ratio (95% Confidence interval) unless otherwise stated.

Model 1: Age, gender, systolic blood pressure, body mass index

Model 2: Model 1 + comorbidity (hypertension, coronary artery disease, atrial fibrillation, chronic obstructive pulmonary disease)

Model 3: Model 2 + medications (angiotensin converting enzyme inhibitor/ angiotensin Ⅱ receptor blocker, beta blocker, statin, SGLT2 inhibitors, GLP-1 receptor agonists)

Model 4: Model 3 + laboratory data (low-density lipoprotein-cholesterol, glycated hemoglobin, estimated glomerular filtration rate measured by CKD-EPI (Chronic Kidney Disease Epidemiology Collaboration))
