# Supplementary material for: Diabetic Retinopathy Severity and Heart Failure Outcomes in Type 2 Diabetes Mellitus
Source: J Diabetes. 2026 Jul 2;18(7):e70235. doi: 10.1111/1753-0407.70235 (PMC13328843; doi:10.1111/1753-0407.70235)
Supplement: Supplementary file 14 — Table S7: Incident heart failure according to diabetic retinopathy severity stratified by insulin use. [file JDB-18-e70235-s012.docx]

**Supplementary Table 7.** Incident heart failure according to diabetic retinopathy severity stratified by insulin use

|  | No apparent (n=13,950) | Mild non-proliferative (n=1,630) | Moderate to severe non-proliferative and proliferative (n=1,875) | P for trend |
| --- | --- | --- | --- | --- |
| **Non-Insulin Users (n=16,522)** | |  |  |  |
| Incident heart failure (n) | 289 | 64 | 92 |  |
| Person-year | 46682.5 | 5852.3 | 6043.8 |  |
| Incidence rate (per 1,000 person-year) | 6.19 (5.49–6.96) | 10.94 (8.42–13.97) | 15.23 (12.29–18.68) |  |
| Crude Incidence rate ratio | Reference | 1.77 (1.35–2.33) | 2.46 (1.96–3.09) | <0.001 |
| Adjusted Incidence rate ratio | Reference | 1.52 (1.15–2.01) | 1.68 (1.32–2.13) | <0.001 |
| Hazard ratio |  |  |  |  |
| Model 1 | Reference | 1.69 (1.29–2.22) | 2.51 (2.00–3.16) | <0.001 |
| Model 2 | Reference | 1.67 (1.27–2.20) | 2.35 (1.86–2.97) | <0.001 |
| Model 3 | Reference | 1.61 (1.22–2.13) | 2.18 (1.71–2.77) | <0.001 |
| Model 4 | Reference | 1.50 (1.14–1.99) | 1.66 (1.30–2.12) | <0.001 |
| **Insulin Users (n=5,251)** | |  |  |  |
| Incident heart failure (n) | 70 | 36 | 76 |  |
| Person-year | 8698.2 | 1731.1 | 3015.8 |  |
| Incidence rate (per 1,000 person-year) | 8.05 (6.29–10.15) | 20.79 (14.57–28.78) | 25.21 (19.84–31.54) |  |
| Crude Incidence rate ratio | Reference | 2.58 (1.74–3.83) | 3.13 (2.28–4.30) | <0.001 |
| Adjusted Incidence rate ratio | Reference | 1.72 (1.14–2.60) | 1.85 (1.32–2.60) | <0.001 |
| Hazard ratio |  |  |  |  |
| Model 1 | Reference | 2.36 (1.59–3.51) | 3.38 (2.46–4.65) | <0.001 |
| Model 2 | Reference | 2.30 (1.54–3.43) | 3.12 (2.26–4.30) | <0.001 |
| Model 3 | Reference | 2.15 (1.43–3.24) | 2.78 (2.01–3.85) | <0.001 |
| Model 4 | Reference | 1.70 (1.12–2.57) | 1.87 (1.33–2.63) | <0.001 |

The data are expressed as ratio (95% Confidence interval) unless otherwise stated.

Model 1: Age, gender, systolic blood pressure, body mass index

Model 2: Model 1 + comorbidity (hypertension, coronary artery disease, atrial fibrillation, chronic obstructive pulmonary disease)

Model 3: Model 2 + medications (angiotensin converting enzyme inhibitor/ angiotensin Ⅱ receptor blocker, beta blocker, statin, SGLT2 inhibitors, GLP-1 receptor agonists)

Model 4: Model 3 + laboratory data (low-density lipoprotein-cholesterol, glycated hemoglobin, estimated glomerular filtration rate measured by CKD-EPI (Chronic Kidney Disease Epidemiology Collaboration)

Abbreviations: eGFR= estimated glomerular filtration rate.
