# Supplementary material for: Diabetic Retinopathy Severity and Heart Failure Outcomes in Type 2 Diabetes Mellitus
Source: J Diabetes. 2026 Jul 2;18(7):e70235. doi: 10.1111/1753-0407.70235 (PMC13328843; doi:10.1111/1753-0407.70235)
Supplement: Supplementary file 15 — Table S8: Mediation analysis of renal factors in the association between diabetic retinopathy severity and heart failure. [file JDB-18-e70235-s001.docx]

**Supplementary Table 8.** Mediation analysis of renal factors in the association between diabetic retinopathy severity and heart failure

| **Effect / Component** | **eGFR** | **P value** | **UACR stage** | **P value** |
| --- | --- | --- | --- | --- |
| **Total Effect** | **Path coefficient (95% CI)** |  | **Path coefficient (95% CI)** |  |
| Mild non-proliferative vs No apparent → AHF | 0.03873 (0.02844 to 0.04901) | <0.001 | 0.03873 (0.02844 to 0.04901) | <0.001 |
| Moderate to severe non-proliferative and proliferative vs No apparent → AHF | 0.08769 (0.07836 to 0.09703) | <0.001 | 0.08769 (0.07836 to 0.09703) | <0.001 |
| **Direct Effect** |  |  |  |  |
| Mild non-proliferative vs No apparent → AHF | 0.03499 (0.02486 to 0.04512) | <0.001 | 0.02879 (0.01670 to 0.04089) | <0.001 |
| Moderate to severe non-proliferative and proliferative vs No apparent → AHF | 0.06980 (0.06054 to 0.07906) | <0.001 | 0.07348 (0.06274 to 0.08423) | <0.001 |
| **Indirect Effect (Mediation)** |  |  |  |  |
| Mild non-proliferative vs No apparent → mediator → AHF | 0.00386 (0.00193 to 0.00580) | <0.001 | 0.00068 (−0.00044 to 0.00179) | 0.234 |
| Moderate to severe non-proliferative and proliferative vs No apparent → mediator → AHF | 0.01749 (0.01537 to 0.01962) | <0.001 | 0.01128 (0.00917 to 0.01340) | <0.001 |
| **Mediation Component** |  |  |  |  |
| Mild non-proliferative vs No apparent → mediator | −1.87829 (−2.81010 to −0.94648) | <0.001 | 0.02935 (−0.01870 to 0.07741) | 0.231 |
| mediator → AHF | −0.00206 (−0.00220 to −0.00191) | <0.001 | 0.02304 (0.01920 to 0.02687) | <0.001 |
| Moderate to severe non-proliferative and proliferative vs No apparent → mediator | −8.50452 (−9.34849 to −7.66054) | <0.001 | 0.48989 (0.44784 to 0.53194) | <0.001 |

Path coefficients represent unstandardized estimates derived from mediation models. Confidence intervals were computed using the Standard (Delta method). Models were adjusted for age and sex. Diabetic retinopathy was categorized as no diabetic retinopathy, mild nonproliferative diabetic retinopathy, and moderate to severe nonproliferative diabetic retinopathy or proliferative diabetic retinopathy.

Abbreviations: eGFR= estimated glomerular filtration rate; UACR = urine albumin-to-creatinine ratio.
